# Club cell‐specific telomere protection protein 1 (TPP1) protects against tobacco smoke‐induced lung inflammation, xenobiotic metabolic dysregulation, and injurious responses

**DOI:** 10.1096/fba.2023-00115

**Published:** 2024-01-15

**Authors:** Thivanka Muthumalage, Chiara Goracci, Irfan Rahman

**Affiliations:** ^1^ Department of Environmental Medicine, School of Medicine and Dentistry University of Rochester Medical Center Rochester New York USA

**Keywords:** ACD, basal cells, bronchogenic carcinomas, cigarette smoke, club cells, COPD, fibrosis, mainstream smoke, shelterin, TPP1

## Abstract

Inhaling xenobiotics, such as tobacco smoke is a major risk factor for pulmonary diseases, e.g., COPD/emphysema, interstitial lung disease, and pre‐invasive diseases. Shelterin complex or telosome provides telomeric end protection during replication. Telomere protection protein 1 (TPP1) is one of the main six subunits of the shelterin complex supporting the telomere stability and genomic integrity. Dysfunctional telomeres and shelterin complex are associated as a disease mechanism of tobacco smoke‐induced pulmonary damage and disease processes. The airway epithelium is critical to maintaining respiratory homeostasis and is implicated in lung diseases. Club cells (also known as clara cells) play an essential role in the immune response, surfactant production, and metabolism. Disrupted shelterin complex may lead to dysregulated cellular function, DNA damage, and disease progression. However, it is unknown if the conditional removal of TPP1 from Club cells can induce lung disease pathogenesis caused by tobacco smoke exposure. In this study, conditional knockout of Club‐cell specific TPP1 demonstrated the instability of other shelterin protein subunits, such as TRF1, dysregulation of cell cycle checkpoint proteins, p53 and downstream targets, and dysregulation of telomeric genes. This was associated with age‐dependent senescence‐associated genes, increased DNA damage, and upregulated RANTES/IL13/IL33 mediated lung inflammation and injury network by cigarette smoke (CS). These phenomena are also associated with alterations in cytochrome P450 and glutathione transferases, upregulated molecular pathways promoting lung lesions, bronchial neoplasms, and adenocarcinomas. These findings suggest a pivotal role of TPP1 in maintaining lung homeostasis and injurious responses in response to CS. Thus, these data TPP1 may have therapeutic value in alleviating telomere‐related chronic lung diseases.

## Graphical abstract

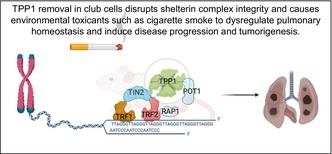

## 1 INTRODUCTION

Tobacco smoking is a risk factor for many respiratory illnesses, such as chronic obstructive pulmonary disease (COPD), asthma, and pulmonary fibrosis. Our lab and others have recognized telomere dysfunction and cellular senescence as potential pathways of respiratory disease mechanisms.^1^, ^2^, ^3^, ^4^, ^5^, ^6^, ^7^, ^8^ Telomere attrition has been associated with ever‐smokers compared to never‐smokers.^9^ Telomeric protein complexes, shelterin and CST (CTC1‐STN1‐TEN1), are integral to telomere length maintenance and chromosomal end‐capping.^10^ Shelterin complex consisting of TRF1, TRF2, Rap1, TIN2, TPP1, and POT1 prevents telomeres from activating DNA damage signaling repair pathways, blocks nonhomologous end joining, and maintains telomeric 3′ overhang.^11^ Our lab has previously shown that cigarette smoke (CS) disrupts telomere protection protein (TPP1) in club cells (also referred to as clara cells).^5^

The role of club cells in pulmonary pathology is not well understood. However, it is well known that these secretoglobin proteins (SCGB) or clara cell secretory protein (CCSP), also known as CC10 and CC16, are present in mucosal epithelia of lungs, specifically in the tracheobronchial region and goblet cells.^12^, ^13^ The primary function of the clara cells/club cells is to secrete surfactant proteins, form epithelial lining fluid, and metabolize any xenobiotic compounds, as clara cells contain most of the P450 enzymes in the lungs. In this study, we utilized a transgenic mouse model TPP1^CreCC10^ where the TPP1 has been conditionally deleted in clara/club cells to understand the role of TPP1 in tobacco smoke‐induced lung pathogenesis.

Tobacco smoke consists of over 7000 chemicals, out of which at least 60 are classified as carcinogens.^14^ Tobacco smoke has particulate and gas phase compounds. The particulate phase includes tar, nicotine, carboxylic acids, phenols, humectants, terpenoids, tobacco‐specific nitrosamines, polycyclic aromatic hydrocarbons (PAH), and catechol. The gas phase comprises volatile organic compounds (VOCs) and semi‐volatile organic compounds (SVOC). These include nitrogen, oxygen, carbon dioxide, carbon monoxide, acetaldehyde, methane, hydrogen cyanide, acetone, acrolein, ammonia, methanol, and hydrogen sulfide. It has been estimated that a puff of tobacco smoke has ~1 × 10^5^ and these reactive oxygen species, such as hydroxyl radicals by additions to C=C bonds, lead to biomolecule adduct formation resulting in oxidative stress and DNA damage.^15^, ^16^, ^17^ Inhalation of these xenobiotics would result in phase 1 metabolism via the CYP enzyme complex in the lungs.

We hypothesize that conditional removal of TPP1 from the club cell shelterin complexes would cause shelterin/CST dysfunction, DNA damage, cellular senescence, and cellular dysfunction in response to CS. This would result in hampered surfactant protein production, mucosal immunity, and metabolism, leading to lung pathogenesis such as lung inflammation and injury, COPD or asthma phenotype, and carcinoma formations.

## 2 METHODS

### 2.1 Ethics statement: institutional biosafety and animal protocol approval

The experimental protocols were approved by the University of Rochester Institutional Biosafety Committee (study approval number: Rahman/102054/09–167/07–186; identification code: 07–186). The mouse study was approved by the University Committee on Animal Research (UCAR) Committee of the University of Rochester, Rochester, NY (UCAR protocol 102,204/UCAR‐2007‐070E), and other methods used were described recently.^57^

### 2.2 Animals

TPP1^flox^ mice (B6.Cg‐Acdtm1.1Cek/J) with adrenocortical dysplasia (Acd) gene flanked at *loxP* sites (also referred throughout the manuscript as TPP1^fl/fl^ or TPP1 FLOX) generated by Dr. Catherine Keegan (University of Michigan Medical School) was bred with CreCC10 mice.^18^ Resulted progeny with clara cell (club cell) specific TPP1 knockout (TPP1^CreCC10^ CreCC10 also referred throughout the manuscript as TPP1 CreCC10).

For in vivo chronic mainstream CS exposures, adult (2‐7‐months old) adult male and female TPP1^flox^ and TPP1^CreCC10^mice (body weight ∼25 g) were bred and housed at the University of Rochester vivarium under normal light and dark cycles and ad libitum feeding according to the Institutional Animal Care and Use Committee (IACUC)/University Committee on Animal Resources (UCAR) guidelines. For the acute (10‐day) mainstream CS exposures, 6–17‐month‐old TPP1^flox^ and TPP1^CreCC10^ mice were used. To assess age‐dependent effects in wildtype mice, C57BL/6J mice of approximately three different age groups, 2‐, 6‐, and 9‐ month‐old mice were housed at the university vivarium in accordance to the UCAR guidelines.

### 2.3 Mainstream tobacco smoke exposure

TPP1 Flox and TPP1 CreCC10 mice were exposed to mainstream tobacco smoke (also referred to as cigarette smoke or CS) for 4 months (chronic); 2 h/day, 5 days a week, with approximately ~200–250 mg/m^3^ total particular matter (TPM) 3R4F research grade cigarettes (University of Kentucky) according to the Federal Trade Commission protocol puffing regimen (1 puff/min, 2 s duration, and 35 mL puff) using the CSM2072i Baumgartner Jaeger (CH technologies) mainstream smoking machine. Twenty‐four hours after the last smoke exposure, mice were anesthetized for lung function parameters and then euthanized for tissue collection.

The effects of acute exposure to tobacco smoke on the shelterin complex were also assessed by a 10‐day exposure to mainstream CS following the same puffing regimen.

To assess any age‐dependent changes in wildtype mice, C57BL/6J (purchased from Jackson laboratories and then bred in house to age) mice of three age groups were exposed to mainstream smoke for 5 months following the aforementioned regimen.

### 2.4 Measurements of lung function parameters

Lung function parameters were determined using SCIREQ Flexivent apparatus (Montreal, Canada). Briefly, lung compliance (Crs or Cst), lung resistance (Rrs), and tissue elastance (Ers), and tissue damping (G) were measured in mice after anesthetizing the mice.

### 2.5 Bronchoalveolar lavage fluid collection

Upon anesthesia, saline solution was instilled into the trachea and the recovered bronchoalveolar lavage fluid (BALF) was centrifuged. The acellular fraction of the BALF was stored in ‒80°C for the Luminex assay. The pelleted cells were then used for flow cytometry analysis to obtain differential cell counts.^57^

### 2.6 Inflammatory mediators by Luminex assay

The BALF was used with Bio‐Rad 23‐plex‐Group I kit to quantify secreted inflammatory mediators (Bio‐Rad Catalog No. M60009RDPD) according to the manufacturer's instructions. The concentrations of each analyte were compared to the unexposed air group and the analytes that showed significant differences were reported.

### 2.7 Flow cytometry analysis

To count total cells and assess cell viability, acridine orange/propidium iodide (AO/PI) staining was used. The cells were stained with CD45, F4/80, Ly6B.2, CD4, and CD8, cell surface markers to determine macrophages, neutrophils, and T‐cell lymphocytes. Sample acquisition was performed using a Guava easyCyte 8 flow cytometer (Luminex). Data analysis was performed using GuavaSoft 3.3.^57^

### 2.8 Immunoblot analyses

Lung homogenate samples were used for BCA assay (Thermo Scientific, Cat No. 23227) to quantify total protein, and used for SDS polyacrylamide gels (SDS‐PAGE), probed with 1:1000 Chk1 (Abcam, ab585567), 1:1000 OBFC1 (Santa Cruz, sc376450), TRF1 (Abcam, ab1423), and ZBTB48/TZAP (Abcam, ab225935) primary antibodies overnight at 4°C. Goat Anti‐Rabbit IgG (H + L) secondary antibody was used at a dilution of 1:5000. Same blots were stripped with stripping buffer, washed, and blocked and then probed with β‐actin‐HRP (Abcam, ab20272) as the loading control. Target proteins were normalized against loading control from corresponding samples. Blot images were captured by the Bio‐Rad Chemi Doc imaging system and analyzed by Image Lab software (Bio‐Rad). Full blots are shown in supplementary figures (Figures S5–S8).

### 2.9 Cellular senescence activity assay

Senescence associated‐β‐galactosidase (SA‐β‐gal) activity in lung homogenates was quantified (ENZO, Cat# ENZ‐KIT129) as per the manufacturer's instructions. Briefly, 50 μL of homogenate was added to 2× assay buffer and incubated for 2.5 h before adding the stop solution and measuring the fluorescence at 360 nm ex/465 nm em. Activity was normalized to total protein in respective samples by the Pierce BCA assay (Thermo Scientific, Cat# 23225).

### 2.10 Gene expression profiling by NanoString sprint profiler

RNA samples were quantified through NanoDrop spectrophotometer (ND‐1000, NanoDrop Technologies), used for NanoString analysis. Premade NanoString code sets for mouse senescence, telomere, DNA damage, and cancer associate genes were used.^57^ Gene expressions were assessed after quality check and normalization using nSolver 4.0 software. Significantly upregulated genes (*p* < 0.05) curated and Venn diagram was prepared using https://bioinformatics.psb.ugent.be/webtools/Venn/.

### 2.11 Proteomics analysis

Approximately 20 mg of snap‐frozen mouse lungs were tested for suitability (no blood contamination) by hemoglobin SDS‐PAGE and the provided Max Quant Log2 fold change values were provided by the University of Rochester proteomics core facility. Subsequently, Log_2_ fold‐change data for TPP1 Flox and CreCC10 mice for air group and tobacco smoke exposed groups were uploaded to QIAGEN Ingenuity Pathway Analysis (IPA) to determine affected biological functions and to identify pathway networks.^19^

### 2.12 Lung histopathology assessment

#### 2.12.1 Hematoxylin eosin staining

Mouse lungs were inflated with 1% low‐melting agarose at 25 cm H_2_O. Formalin fixed lungs were then paraffin embedded with mid‐sagittal sections. Four‐micrometer sections were then stained with hematoxylin and eosin (H&E) stain. Randomly selected six or more images per slide were captured. Mean linear intercept (Lm) was determined by MetaMorph software (Molecular devices) on images captured at 10× magnification with pixel size 7.4 μm. Air space enlargement was compared between TPP1 FLOX and TPP1 CreCC10 strains.

#### 2.12.2 Gomori trichrome staining

The amount of collagen present in the lungs of different experimental groups was quantified by Gomori's trichrome staining using Thermo Fisher Scientific kit (Cat# 87020). Briefly, lung sections (4 μm) were deparaffinized, rehydrated and rinsed with tap water. The sections were then placed in Bouin's solution at 56°C for 1 h and washed with water to remove excess yellow staining. Next, working Weigert's Iron Hematoxylin Stain was applied for 10 min, then removed by washing with water for 10 min. Thereafter, the sections were immersed in Trichrome Stain for 15 min and immediately soaked in acetic acid 1% for 1 min. Finally the sections were rinsed with water, dehydrated, and mounted by Permount (UN1294, Fisher Chemical) for microscopy observation. Ten pictures (20× magnification) have been taken for each sample by Nikon microscopy (Eclipse Ni‐U), and the amount of collagen has been quantified by Color Deconvolution via FIJI ImageJ.

### 2.13 Statistical analysis

The statistical differences between air and mainstream tobacco smoke exposed TPP1^fl/fl^ TPP1^CreCC10^ mouse groups were analyzed through *t*‐tests, one‐way ANOVA, and two‐way ANOVA in GraphPad Prism software (version 9). Results were presented as the mean ± SEM. A *p*‐value of <0.05 was considered significant.

## 3 RESULTS

### 3.1 Exposure to mainstream CS resulted in an inflammatory cell influx in both TPP1 Flox and TPP1 CreCC10 mice

**FIGURE 1:**
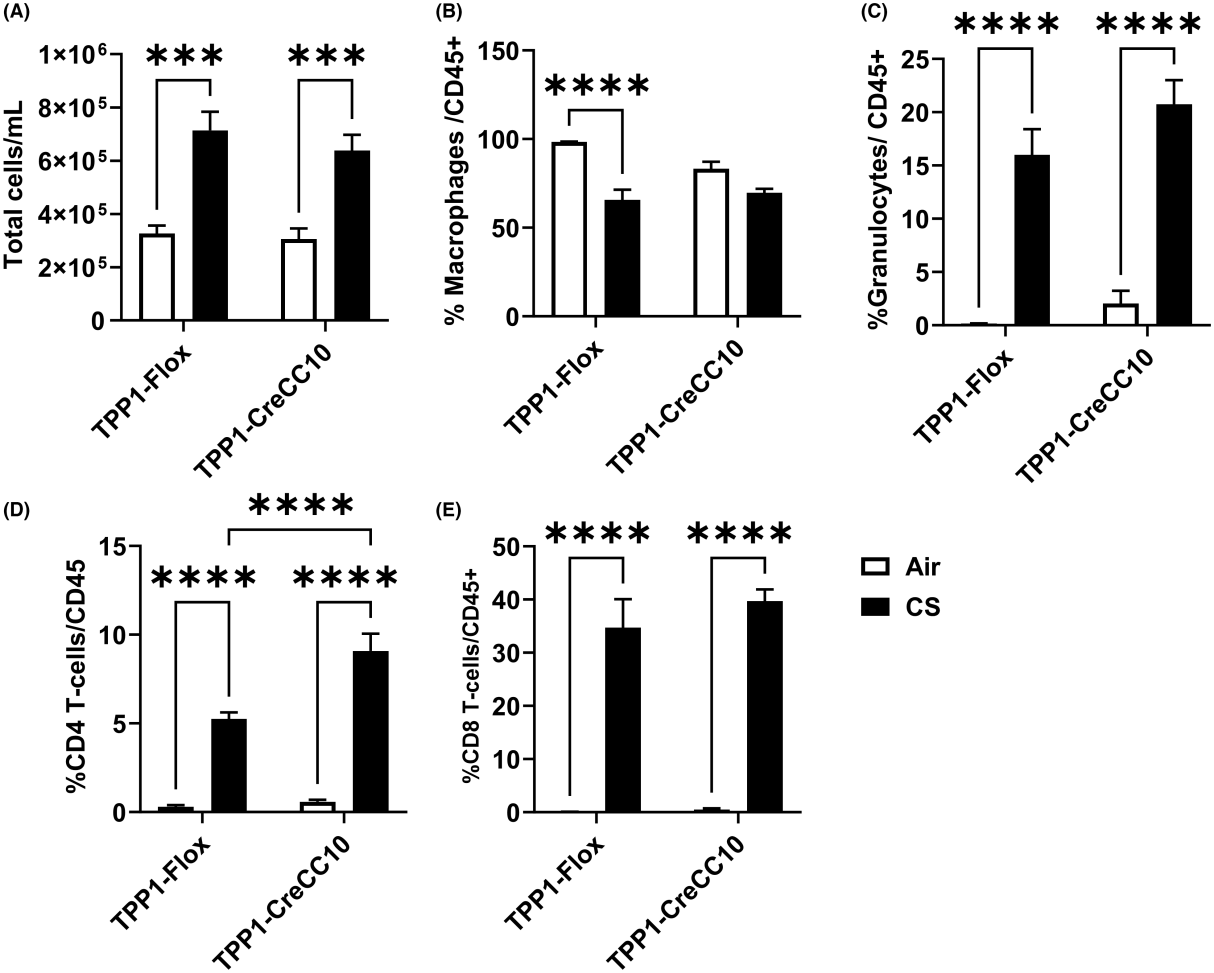
Exposure to mainstream tobacco smoke induced inflammatory cell influx in bronchoalveolar lavage. TPP1‐Flox and TPP1‐CreCC10 mice exposed to mainstream smoke (~200 mg/m TPM; 4 months). After 24 h postexposure, mice were euthanized, and BALF was used for differential cell counts using flow cytometry. (A) Total cell counts per milliliter in BALF were determined by AO/PI staining using a cellometer. Percentage of leukocyte populations were identified from CD45+ parent population with appropriate compensation controls. (B) Percentage of F4/80+ macrophages (C) Percent LY6B.2 granulocytes (D), Percent CD4 T‐lymphocytes lymphocytes (E) Percent CD8 T‐lymphocytes were determined by flow cytometry. (*N* = 6–8/group, two‐way ANOVA, ***p < 0.001, ****p < 0.0001 vs. Air and indicated groups). AO/PI, acridine orange/propidium iodide; BALF, bronchoalveolar lavage fluid; TPM, total particular matter; and TPP1, telomere protection protein 1.

Upon exposure to chronic mainstream CS exposure, BALF total cell number was significantly increased nearly doubled in both TPP1 Flox and TPP1 CreCC10 mice (Figure 1A). Out of the CD45+ parent population, F4‐80+ macrophage count decreased in both Flox and CreCC10 strains (Figure 1B). Ly6B+ neutrophils were significantly increased up to 16% and 21% in Flox and CreCC10 mice, respectively (Figure 1C). CD4+ T_h_ lymphocyte count was significantly increased up to 5% and 9% in Flox and CreCC10 mice, respectively (Figure 1D). CD8+ T_c_ cells were increased up to 35% and 40% in Flox and CreCC10 mice, respectively (Figure 1E). Overall, both flox and CreCC10 groups demonstrated similar trends in cell influx, but the increase in CD4 count was significantly greater in the CreCC10 group.

Upon acute exposure (10 days) to CS, we observed a significant total cell influx (*p* < 0.01) in TPP1 Flox mice compared to the TPP1 CreCC10 group (Figure S1). The increase was mainly attributed to the infiltration of macrophages as the total macrophage count in BALF was significantly elevated after the exposure in the TPP1 Flox mice compared to the CreCC10 group (Figure S1). Macrophages, neutrophils, and CD4‐lymphocyte counts were increased in both TPP1 Flox and CreCC10 groups upon exposure, but the increase in neutrophil and CD4 increases were significant in TPP1 CreCC10 group. (Figure S1).

### 3.2 Exposure to mainstream smoke differentially altered inflammatory mediators in both TPP1 Flox and TPP1 CreCC10 mice

**FIGURE 2:**
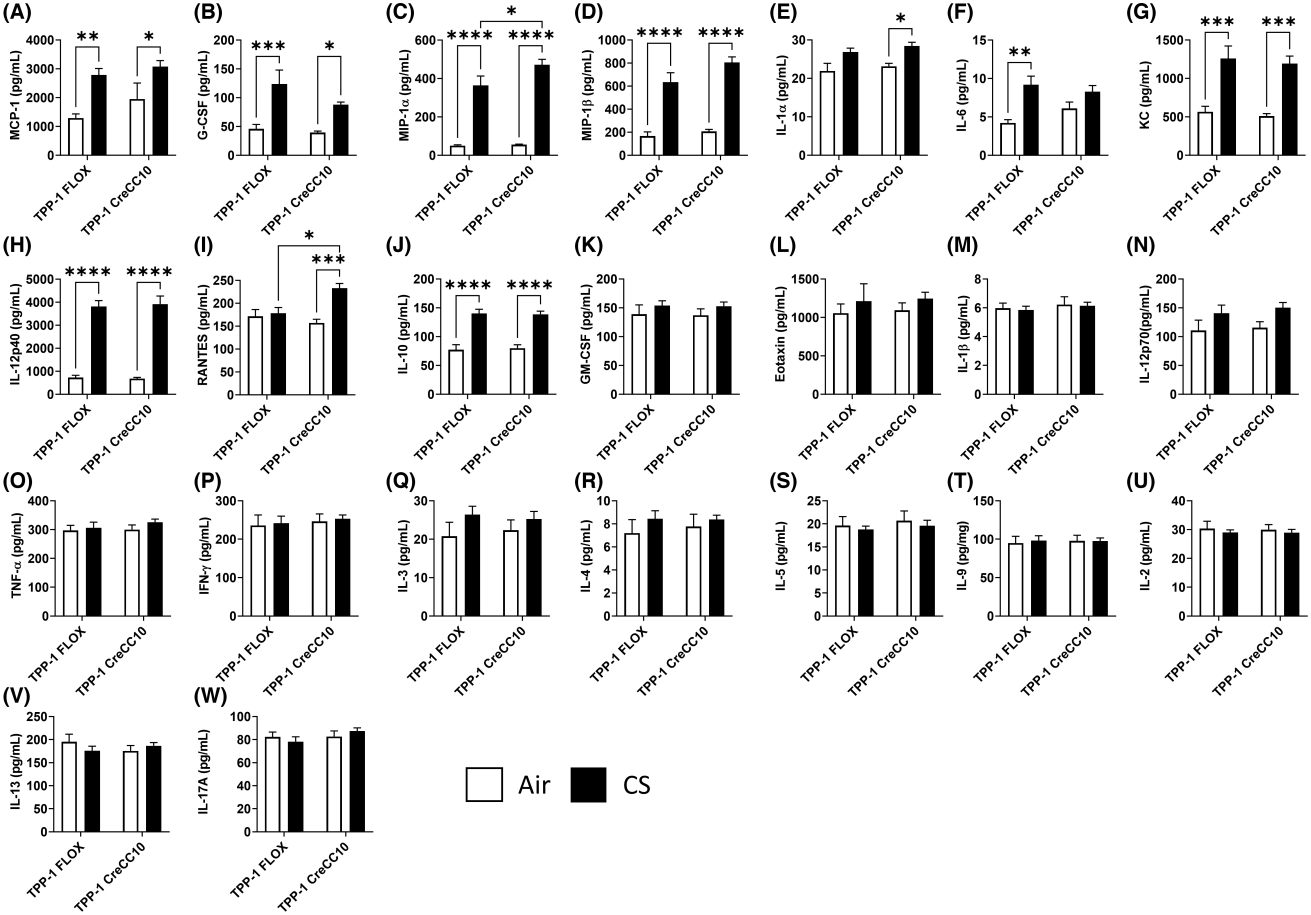
Exposure to mainstream tobacco smoke elicited a differential cytokine response in bronchoalveolar lavage. TPP1‐Flox and TPP1‐CreCC10 mice exposed to mainstream smoke (~200 mg/m TPM; 4 months). After 24 h postexposure, mice were euthanized, and BALF collected and secreted cytokines were quantified by Luminex including (A) *MCP‐1*, (B) *G‐CSF*, (C) *MIP1‐α*. (D) *MIP‐1β*, (E) *IL‐1α*, (F) *IL‐6*, (G) *KC*, (H) *IL‐12p40*, (I) *RANTES*, (J) *IL‐10*, (K) *GM‐CSF*, (L) *Eotaxin*, (M) *IL‐1β*, (N) *IL‐12p70*, (O) *TNF‐α*, (P) *IFN‐γ*, (Q) *IL‐3*, (R) *IL‐4*, (S) *IL‐5*, (T) *IL‐9*, (U) *IL‐2*, (V) *IL‐13*, and (W) *IL‐17A*. (*N* = 6–8/group, two‐way ANOVA, *p < 0.05, **p < 0.01, ***p < 0.001, ****p < 0.0001 vs. Air and indicated groups). BALF, bronchoalveolar lavage fluid, TPM, total particular matter; and TPP1, telomere protection protein 1.

In both TPP1 flox and TPP1 CreCC10 mouse BALF, MCP‐1 (Figure 2A), G‐CSF (Figure 2B), MIP‐1α (Figure 2C), MIP‐1β (Figure 2D), KC (Figure 2G), IL12p40 (Figure 2H), and IL‐10 (Figure 2J) were significantly increased upon exposure to chronic mainstream smoke. The increase in MIP‐1α was significant in the CreCC10 group compared to the TPP1 Flox counterparts. IL‐1α (Figure 2E) and RANTES (Figure 2I) were significantly elevated only in TPP1 CreCC10 group compared to the air group. GM‐CSF, eotaxin, IL‐1β, IL12p70, TNFα, IFNγ, IL‐3, IL‐4, IL‐5, IL‐9, IL‐2, IL‐13, and IL‐17A levels were unaffected by the exposure in both strains (Figure 2K–W). Interestingly, IL6 levels were significantly increased only in TPP1 Flox smoke‐exposed mice compared to their unexposed counterparts (Figure 2F). RANTES levels were significantly increased in TPP1‐CreCC10 mice postexposure to CS compared to unexposed mice while no change was observed in TPP1‐FLOX counterparts (Figure 2I).

Acute exposure to CS significantly elevated IL6 and eotaxin levels TPP1 CreCC10 mice compared to the TPP1 Flox mice (Figure S2). While IL13 was significantly attenuated in TPP1 Flox mice, no changes were observed in the CreCC10 group (Figure S2). Inflammatory mediators, MCP‐1, KC, and MIP1α, were significantly increased in both TPP1 Flox and TPP1 CreCC10 groups (Figure S2).

### 3.3 Exposure to mainstream tobacco smoke exhibited mild restrictive lung function in TPP1 CreCC10 mice and age‐dependent effects in wild type mice

**FIGURE 3:**
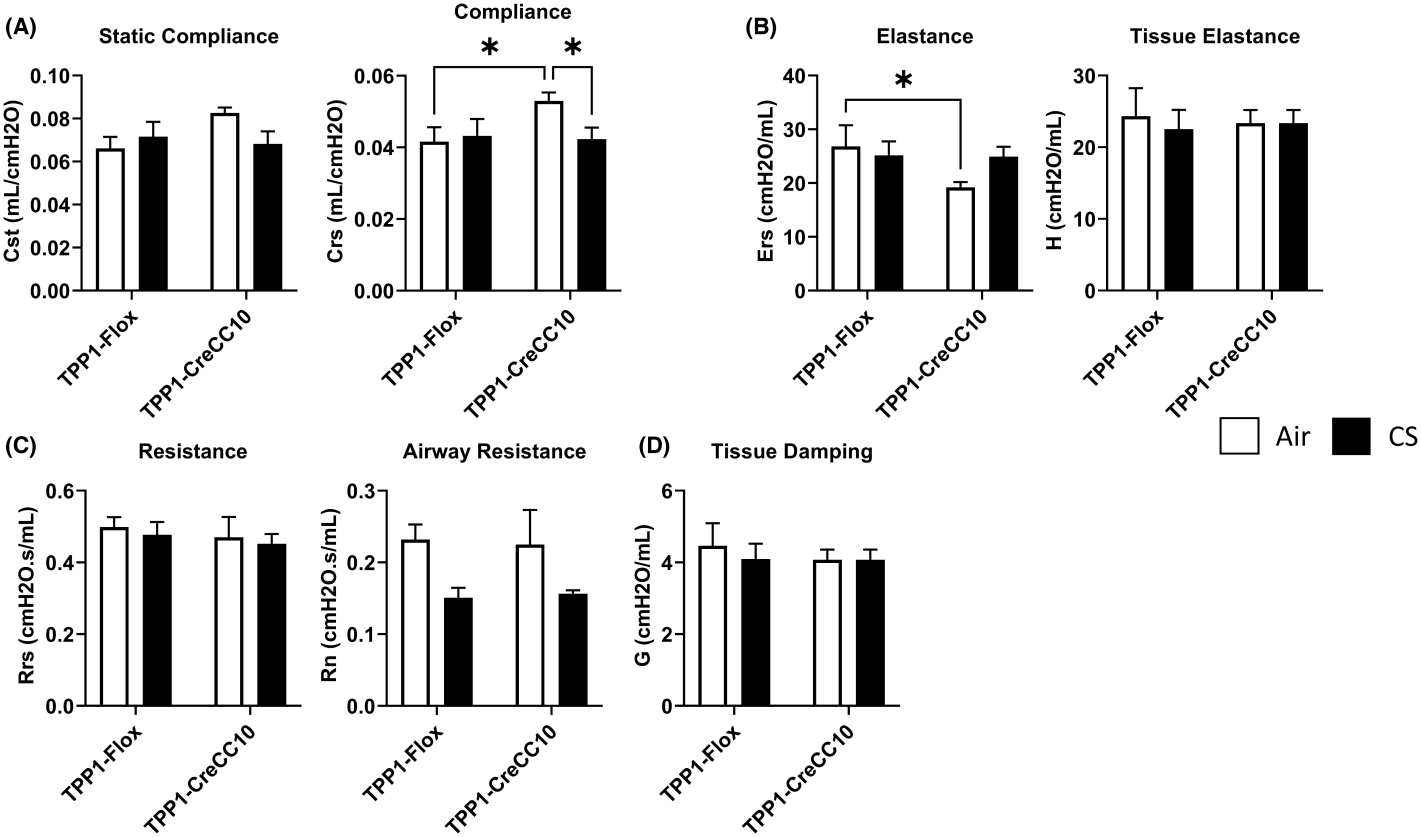
Exposure to mainstream tobacco smoke did not alter lung mechanics parameters in TPP1 Flox with marginally increased elastance in TPP1 CreCC10 mice. TPP1‐Flox and TPP1‐CreCC10 mice exposed to mainstream smoke (~200 mg/m TPM; 4 months). After 24 h postexposure, mice were anesthetized and lung function parameters including, (A) compliance, (B) elastance, (C) resistance, and (D) tissue damping were measured by Flexivent. (*N* = 6–8/group, two‐way ANOVA, *p < 0.05 vs. Air and indicated groups). TPM, total particular matter and TPP1, telomere protection protein 1.

After the chronic CS exposure, in adult mice (up to ~7 months), lung mechanics measurements by Flexivent showed significantly reduced lung compliance (Figure 3A) and increased lung elastance (Figure 3B), in TPP1 CreCC10 mice. These parameters were unaffected in TPP1 Flox mice. Further, resistance (Figure 3C) and tissue damping (Figure 3D) remained unaffected in both strains.

In TPP1 Flox and CreCC10 older cohort up to ~15 months, upon exposure to CS, similar to the mature adult group, showed slightly reduced compliance and increased elastance (statistically not significant) in CreCC10 group compared to the Flox mice (Figure S3). However, tissue damping was increased in these older mice.

To further assess the age‐dependent decline of lung function in wildtype mice, we assessed compliance, elastance, and tissue damping of young (~2 months), middle‐aged (~6 months), and older (~9 months) C57BL/6J mice. Young mice had no changes in any of the tested parameters. Though not statistically significant, the middle‐aged (~6 months) showed decreased compliance, increased elastance, and increased tissue damping (Figure S4). In older mice (9 months) we observed slightly increased compliance and decreased elastance tendencies suggesting age‐dependent effects in wild type mice upon chronic exposure to CS (Figure S4).

### 3.4 Mainstream tobacco smoke exposure caused airspace enlargement and increased collagen deposition in wild type mice

**FIGURE 4:**
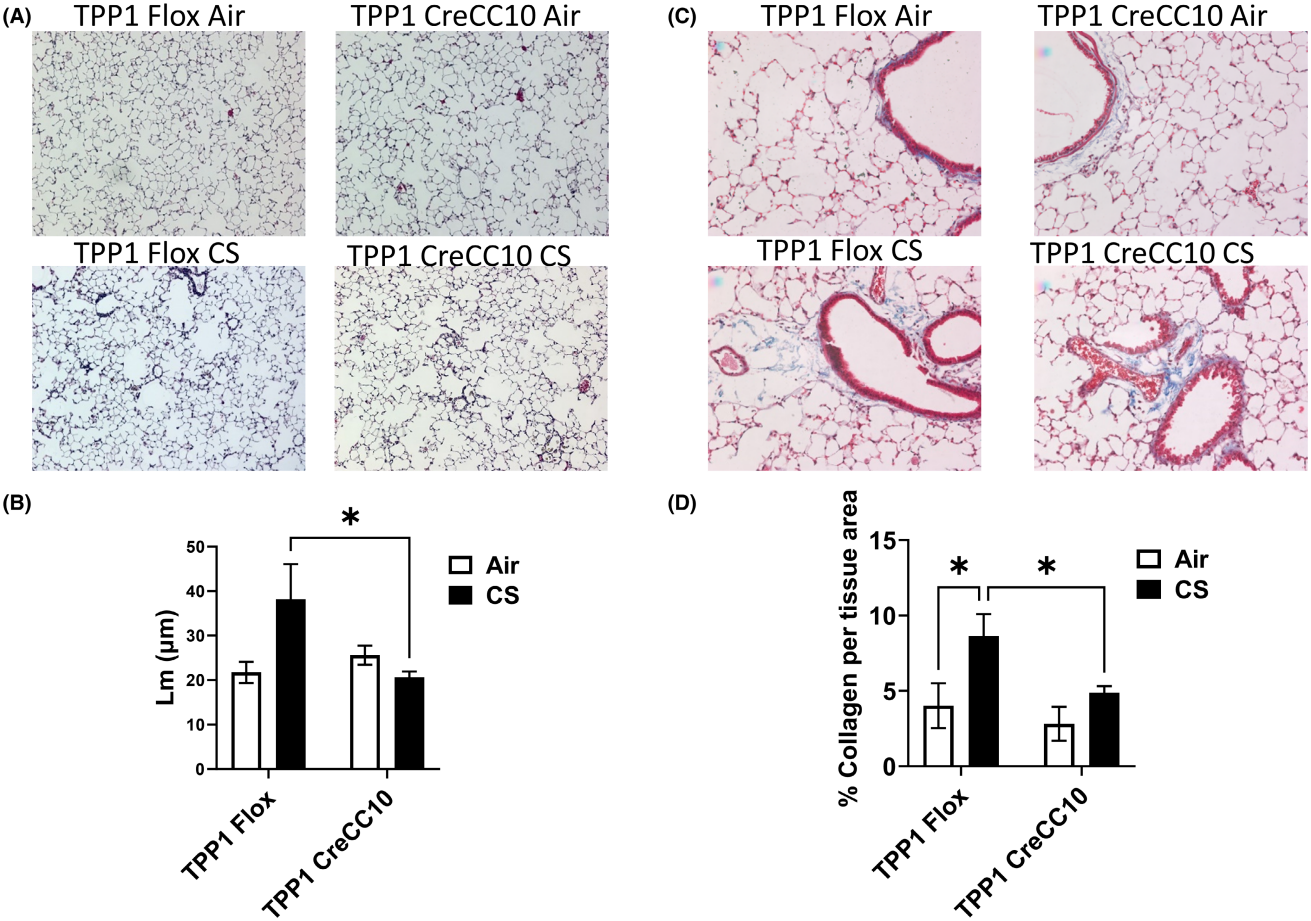
Exposure to mainstream tobacco smoke induced air space enlargement in TPP1 Flox mice and marginal fibrotic changes in TPP1 CreCC10 mice. TPP1‐Flox and TPP1‐CreCC10 mice exposed to mainstream smoke (~200 mg/m TPM; 4 months). After 24 h postexposure, mice were euthanized and large lung lobe was inflated with agarose. Mid‐sagittal Paraffin embedded tissues were sectioned at 4 microns for histological staining. (A) Hematoxylin and eosin (H&E) (B) Air‐space enlargement in H&E stained tissues. (C) Trichrome staining for collaged accumulation (D) collagen deposition. (*N* = 6–8/group, two‐way ANOVA *p < 0.05 vs. indicated group Air and indicated group). TPM, total particular matter and TPP1, telomere protection protein 1.

Exposure to mainstream smoke caused increased Lm values in TPP1 Flox mice compared to TPP1 CreCC10 mice (Figure 4A,B). CS exposure did not cause a significant air space enlargement in TPP1 CreCC10 group (Figure 4B). While CS exposure caused increased collagen deposition in both Flox and CreCC10 groups, significantly increased collagen deposition was also observed in TPP1 Flox mice (Figure 4C,D).

### 3.5 Exposure to chronic mainstream tobacco smoke affected cell cycle, shelterin and CST complexes, and telomere elongation

**FIGURE 5:**
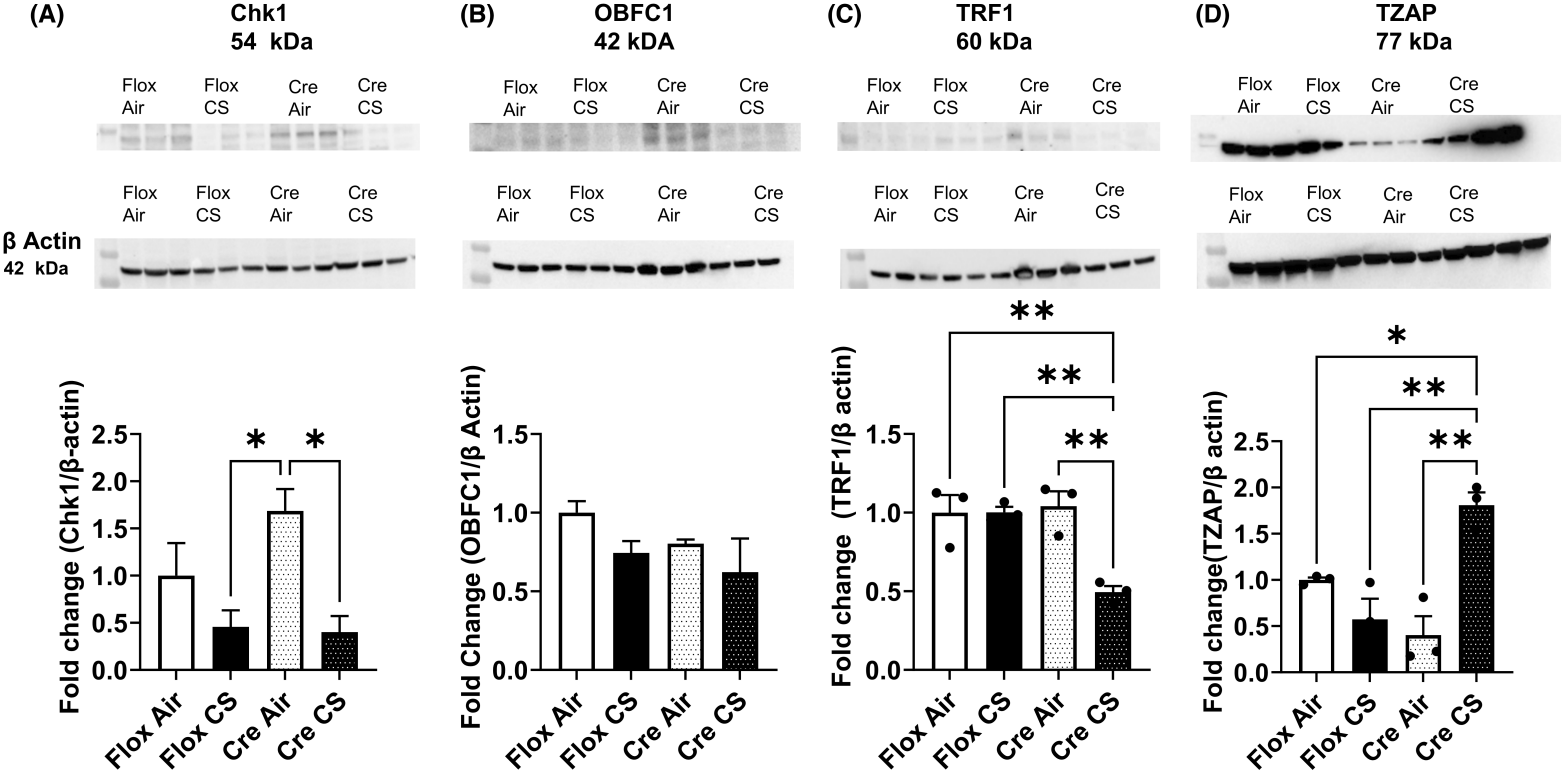
Exposure to mainstream tobacco altered cell cycle and telomere elongation proteins. TPP1‐Flox and TPP1‐CreCC10 mice exposed to mainstream smoke (~200 mg/m TPM; 4 months). After 24 h postexposure, mice were euthanized and cell cycle check point one, CST complex subunit OBFC1, telomeric repeat binding factor 1 (TRF1), and telomeric zinc finger–associated protein abundance were determined in lung tissues by immunoblotting. The same immunoblot was probed with loading control β actin for normalization. (A) Chk1, (B) OBFC1, (C)TF1, and (D) TZAP protein abundance were presented as a fold change compared to TPP1 FLOX air. (*N* = 3/group, *p < 0.05, **p < 0.01 vs. Air and indicated groups). TPM, total particular matter and TPP1, telomere protection protein 1.

Exposure significantly reduced cell cycle regulator at G1/S checkpoint kinase‐1 in TPP CreCC10 mice compared to TPP1 flox mice (Figure 5A). CST complex protein, OBFC1, was not affected by the smoke exposure. However, shelterin complex protein, TRF1, was significantly reduced in TPP1 CreCC10 mice (Figure 5B,C). The reduction in TRF1 in CreCC10 mice was significant compared to the TPP1 flox CS exposed mice (Figure 5C). Telomeric zinc finger–associated protein (TZAP/ZBTB48) was significantly increased in the CS‐exposed TPP1 CreCC10 mice compared to its air group and TPP1 flox counterparts. Full blots are shown in supplementary figures (Figures S5–S8).

### 3.6 Smoke exposure increased susceptibility to senescence in TPP1 CreCC10 mice

**FIGURE 6:**
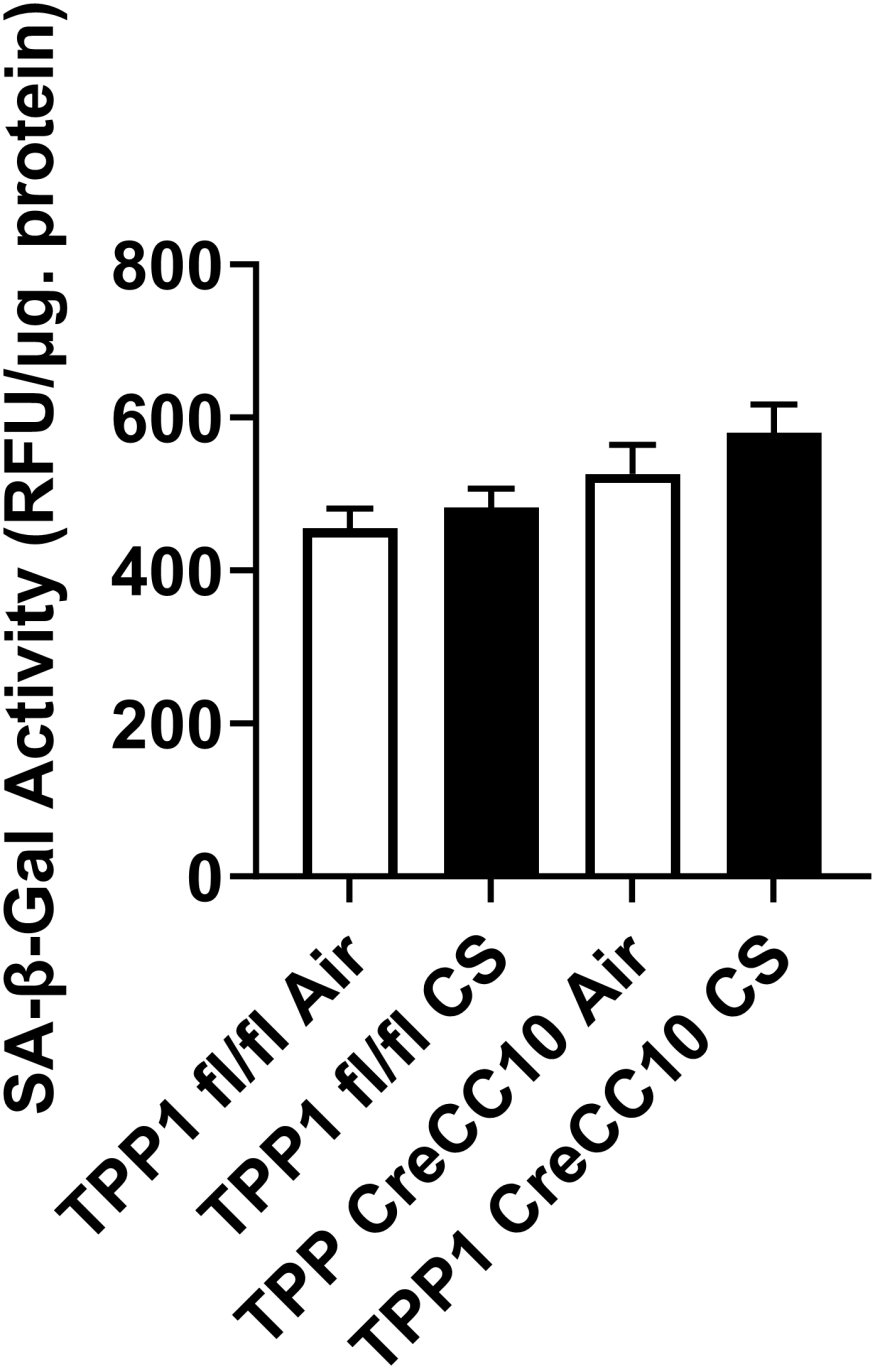
Exposure to mainstream tobacco increased senescence associated beta galactosidase activity in TPP1 CreCC10 mice. TPP1‐Flox and TPP1‐CreCC10 mice exposed to mainstream smoke (~200 mg/m TPM; 4 months). After 24 h postexposure, mice were euthanized and senescence‐associated β‐galactosidase activity was quantified in lung tissues. While SA‐B‐gal activity was not increased in TPP1 FLOX TPP1 CreCC10 CS exposed group showed increased activity compared to the FLOX air group. (*N* = 6/group, two‐way ANOVA). TPM, total particular matter and TPP1, telomere protection protein 1.

Senescence‐associated β‐galactosidase (SA‐β‐gal) activity was measured in mouse lung homogenate. TPP1 flox and Cre mice showed mild increase in SA‐β‐gal activity upon exposure to CS; however, was not significant compared to its unexposed counterparts. This increasing trend suggests susceptibility to cellular senescence in shelterin‐disrupted CS‐exposed mice (Figure 6).

### 3.7 Smoke exposure differentially altered senescence‐associated genes

**FIGURE 7:**
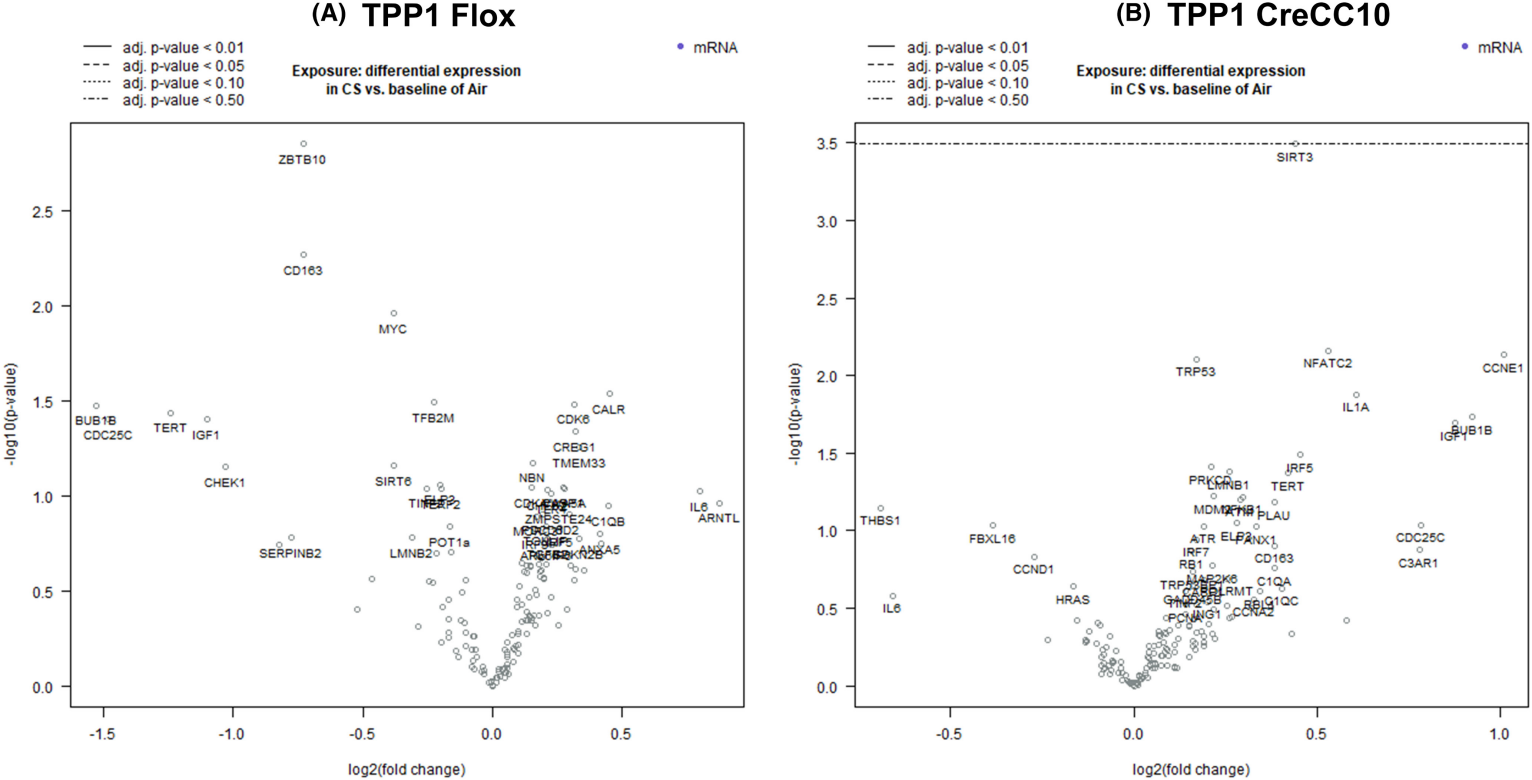
Exposure to mainstream tobacco altered senescence associated genes in TPP1 CreCC10 mice. TPP1‐Flox and TPP1‐CreCC10 mice exposed to mainstream smoke (~200 mg/m TPM; 4 months). After 24 h postexposure, mice were euthanized and senescence associated genes were profiled in lung tissues. (A) Differential expression of senescence genes CS versus Air in TPP1 Flox mice. (B) Differential expression of senescence genes CS versus Air in TPP1 CreCC10 mice. (*N* = 6/group, *p < 0.05, **p < 0.01, ***p < 0.001 vs. Air). TPM, total particular matter and TPP1, telomere protection protein 1.

In smoke exposed TPP1 Flox mouse lungs, Calr was 1.37‐fold increased (*p* < 0.05) while BuB1B, Cdc25c, Tert, Igf1, Check1, Zbtb10, Cd163, and Myc were significantly decreased compared to unexposed mice (Figure 7A). In TPP1 CreCC10 mice, Ccne1, BuB1B, Igf1, Cdc25c, and Il1a genes were significantly upregulated 1.5× more than unexposed counterparts (Figure 7B).

### 3.8 Smoke exposure differentially altered telomere‐associated genes

**FIGURE 8:**
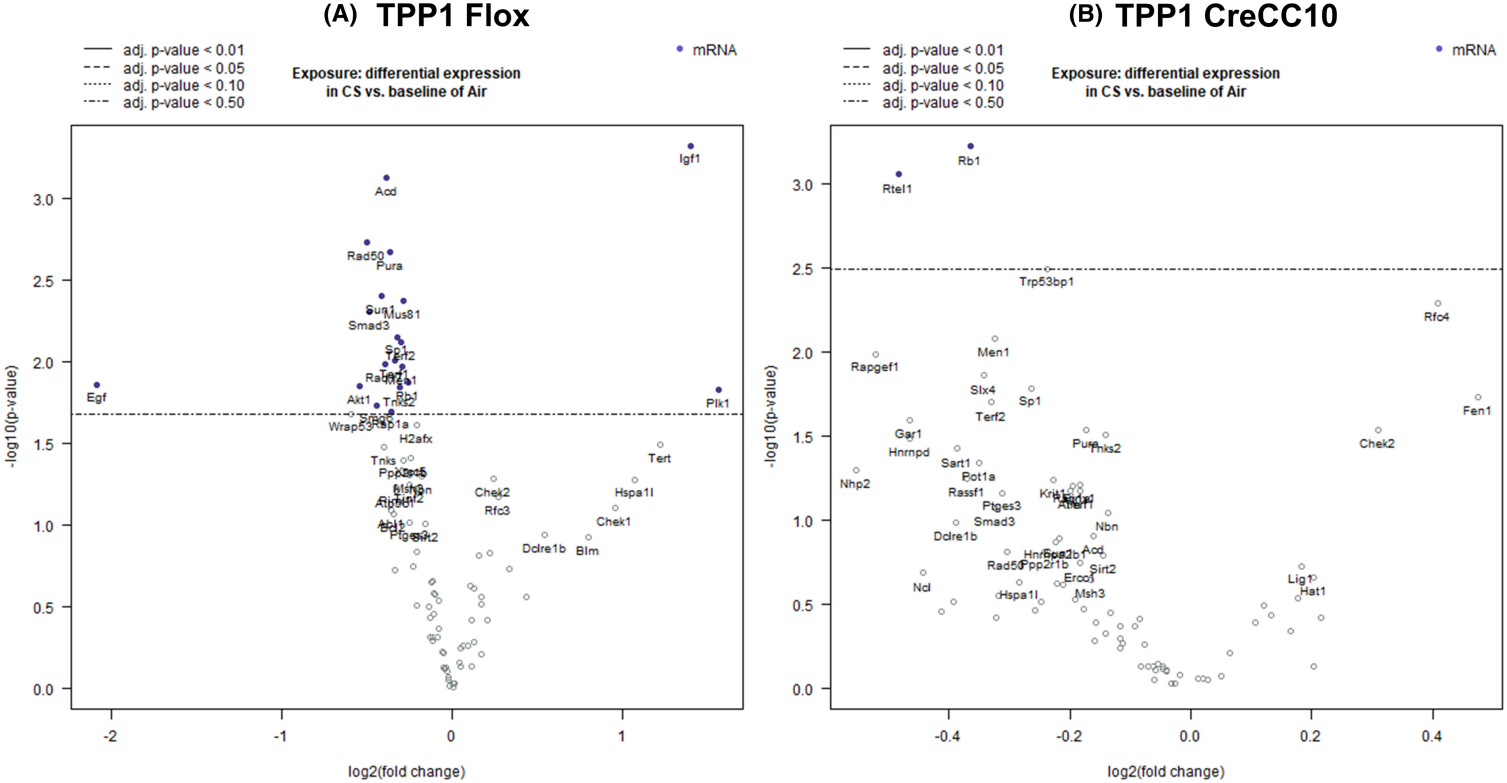
Exposure to mainstream tobacco altered telomere associated genes in TPP1 CreCC10 mice. TPP1‐Flox and TPP1‐CreCC10 mice exposed to mainstream smoke (~200 mg/m TPM; 4 months). After 24 h postexposure, mice were euthanized and telomere associated genes were profiled in lung tissues. (A) Differential expression telomere genes CS versus Air in TPP1 Flox mice. (B) Differential expression of telomere genes CS versus Air in TPP1 CreCC10 mice. (*N* = 6/group, *p < 0.05 **p < 0.01, ***p < 0.001 vs. Air). TPM, total particular matter and TPP1, telomere protection protein 1.

In smoke exposed TPP1 Flox mice, Plk1 and Igf1 levels were increased more than 2.5‐folds compare the unexposed mice (*p* < 0.001). In TPP1 CreCC10 mice, Fen1, Rfc4, and Chek2 were significantly increased and Nhp2, Rapgef1, Rtel1, Gar1, Hnrnpd, Sart1, Rb1, Pot1a, Slx4, Terf2, Men1, Sp1, Trp53bp1, Krit1, Pura, and Tnks2 were significantly decreased compared to unexposed mice (Figure 8A,B).

### 3.9 Smoke exposure differentially altered DNA‐damage‐associated genes

In smoke exposed TPP1‐flox mice, Chek1, Rad51, Neil2, Brip1, Cry1, and Pms1 were greater than 1.5× upregulated (*p* < 0.05) while Dmc1, Cdkn1am Rad50, Xpc, Ppp1r15a, Rad23a, Ercc6, Ercc2, Xrcc5, Pms2, H2afx, and Top3b were significantly downregulated (*p* < 0.05).

**FIGURE 9:**
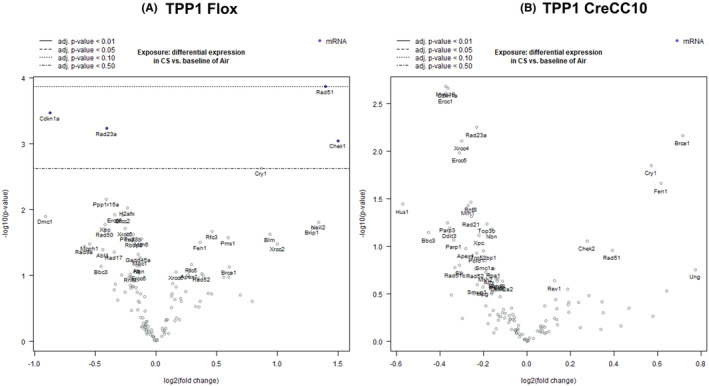
Exposure to mainstream tobacco altered DNA damage‐associated genes in TPP1 CreCC10 mice. TPP1‐Flox and TPP1‐CreCC10 mice exposed to mainstream smoke (~200 mg/m TPM; 4 months). After 24 h postexposure, mice were euthanized and DNA damage associated genes were profiled in lung tissues. (A) Differential expression of DNA damage genes CS versus Air in TPP1 Flox mice. (B) Differential expression of DNA damage related genes CS versus Air in TPP1 CreCC10 mice. (*N* = 6/group, *p < 0.05, **p < 0.01, and ***p < 0.001 vs. Air). TPM, total particular matter and TPP1, telomere protection protein 1.

In TPP1 CreCC10 mice, Brca1, Fen1, and Cry1 were significantly upregulated (*p* < 0.05) while Hus1, Bbc3, Mms19 Ercc1, Parp3, Cdkn1a, Ddit3, Parp1, Ercc5, Xrcc4, Mlh1, Rnf8, Rad21, and Rad23a were significantly downregulated (*p* < 0.05) (Figure 9A,B).

### 3.10 Smoke exposure differentially altered cancer‐associated genes

**FIGURE 10:**
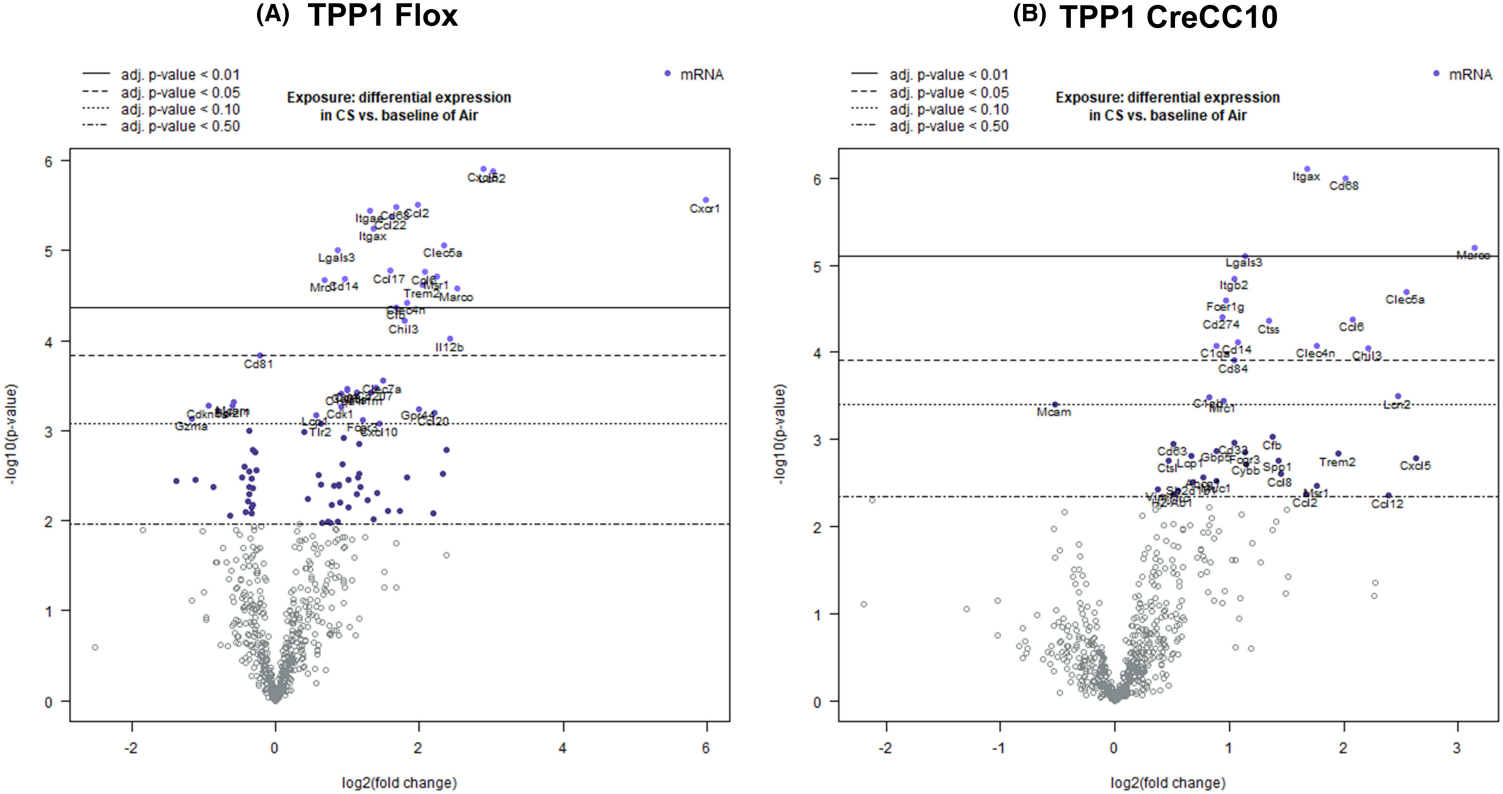
Exposure to mainstream tobacco altered cancer‐associated genes in TPP1 CreCC10 mice. TPP1‐Flox and TPP1‐CreCC10 mice exposed to mainstream smoke (~200 mg/m TPM; 4 months). After 24 h postexposure, mice were euthanized and cancer associated genes were profiled in lung tissues. (A) Differential expression of cancer associated genes CS versus Air in TPP1 Flox mice. (B) Differential expression of cancer genes CS versus Air in TPP1 CreCC10 mice. (*N* = 6/group, *p < 0.05, **p < 0.01, and ***p < 0.001 vs. Air). TPM, total particular matter and TPP1, telomere protection protein 1.

In TPP1 Flox mice, smoke exposure significantly increased Cxcr1 (63×), Lcn2 (8.12×), Cxcl5 (7.42×), Marco (5.76×), Clec5a (5.11×), Msr1 (4.75×), Ccl6 (4.23×), Trem2 (4.11×), Ccl2 (3.93×), Clec4n (3.54×), Chil3 (3.46), Cd68 (3.21×), Cfb (3.21×), Ccl22 (3.08×), Ccl17 (3.03×), Itgax (2.58×), Itgae (2.5×), Cd14 (1.96×), Lgals3 (1.82), and Mrc1 (1.59×). In TPP1 CreCC10 mice, Marco (8.79×), Clec5a (5.86), Lcn2 (5.57), Chil3 (4.63×), Ccl6 (4.22×), Cd68 (4.06), Clec4n (3.39×), Itgax (3.2×), Cfb (2.61×), Ctss (2.56×), Lgals3 (2.2×), Cd14 (2.11×), Itgb2 (2.06×), Cd84 (2.06×), Fcer1g (1.96×), Mrc1 (1.93×), Cd274 (1.91×), C1qa (1.84×), and C1qb (1.77×), while Mcam significantly decreased (Figure 10A,B).

Based on the above gene expression changes in cytokine‐cytokine reception interaction, chemokine signaling pathways, TNF signaling, osteoclast differentiation, Cell adhesion molecules, and pathways in cancer were among the affected identified pathways by the nSolver advanced analysis by the smoke exposure (pathway activation data not shown).

### 3.11 Smoke exposure affected lung proteins associated with DNA replication, chromosome integrity, and tumorigenesis

**FIGURE 11:**
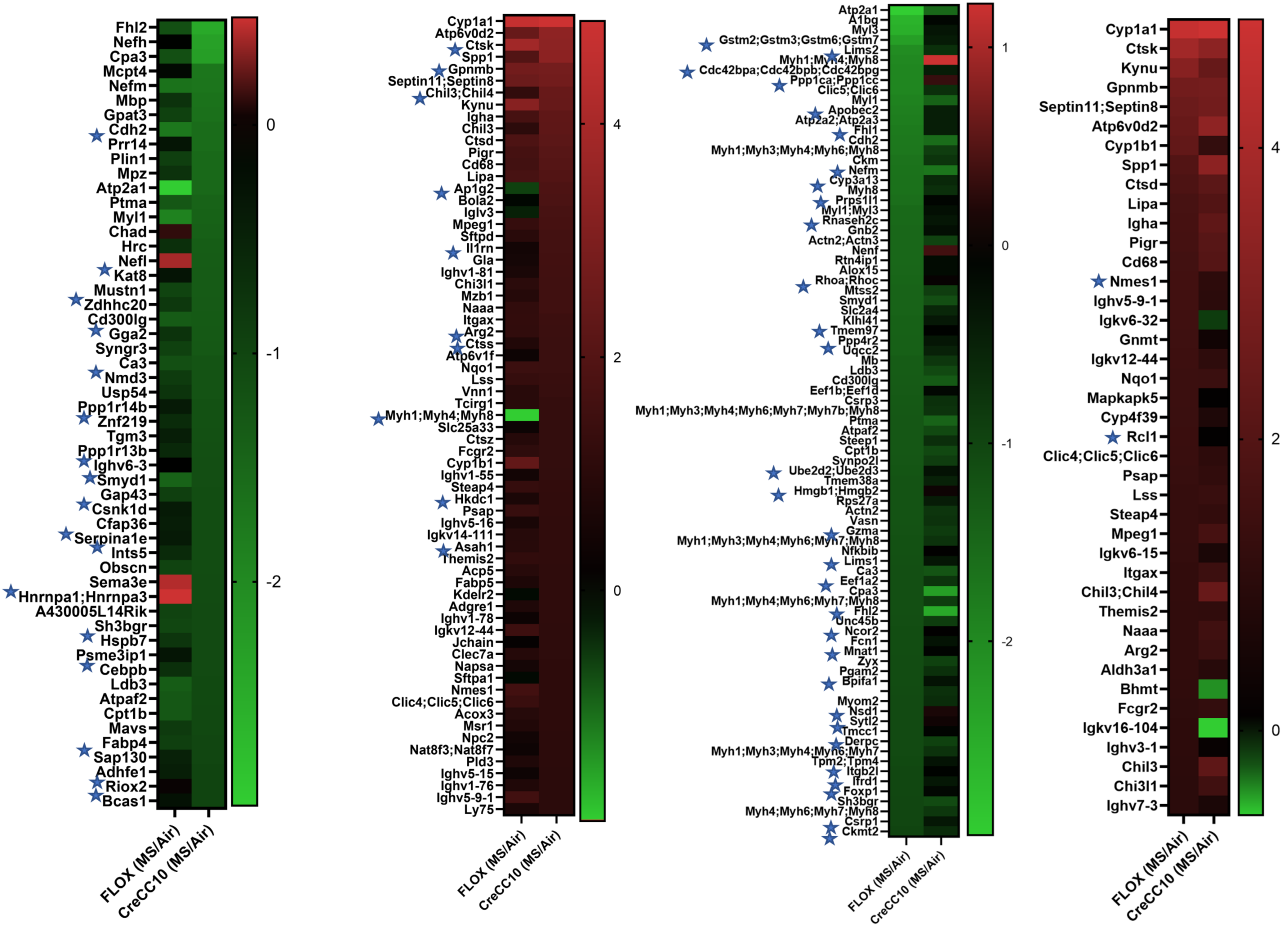
Exposure to mainstream tobacco dysregulated DNA replication, chromosome integrity, and tumorigenesis‐associated proteins in TPP1 CreCC10 mice. TPP1‐Flox and TPP1‐CreCC10 mice exposed to mainstream smoke (~200 mg/m TPM; 4 months). After 24 h postexposure, mice were euthanized and proteomics were performed on lung tissues. Proteins that were greater than 1 Log_2_ Fold identified with *p* < 0.05. Asterisked proteins are related to telomere dysfunction, DNA damage, DNA replication, and tumorigenesis (*N* = 6/group). TPM, total particular matter and TPP1, telomere protection protein 1.

Proteins differentially altered postexposure to mainstream tobacco smoke exposure that were statistically significant in TPP1 Flox and TPP1 CreCC10 were identified and presented as heat maps (Figure 11). Proteins involved in DNA replication, chromosome integrity, and tumorigenesis have been asterisked (Figure 11).

### 3.12 Altered molecular networks by tobacco smoke exposure

**FIGURE 12:**
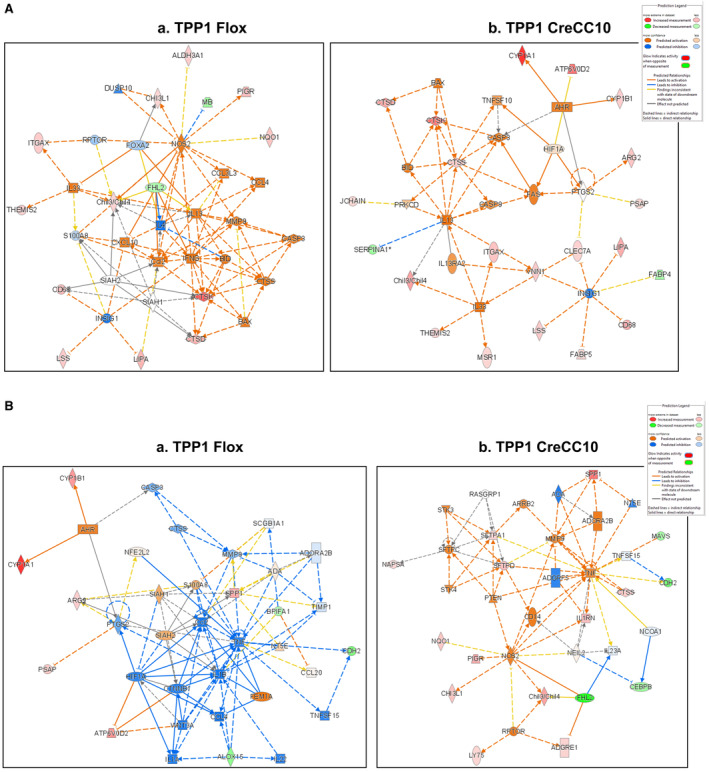
(A) Exposure to mainstream tobacco smoke induced inflammatory response, metabolism, and injury. TPP1‐Flox and TPP1‐CreCC10 mice exposed to mainstream smoke (~200 mg/m TPM; 4 months). After 24 h postexposure, mice were euthanized and proteomics were performed on lung tissues. By Ingenuity Pathway Analysis based on top disease and functions networks identified in (a) TPP1 Flox and (b) TPP1 CreCC10 in which cellular movement, inflammatory response and organismal injuries, and lipid metabolism are affected. (*N* = 6/group). (B) Exposure to mainstream tobacco smoke affected cellular maintenance, movement, cell trafficking, and cell signaling. TPP1‐Flox and TPP1‐CreCC10 mice exposed to mainstream smoke (~200 mg/m TPM; 4 months). After 24 h postexposure, mice were euthanized and proteomics were performed on lung tissues. By Ingenuity Pathway Analysis based on top disease and functions networks identified in (a) TPP1 Flox and (b) TPP1 CreCC10 in which cellular cell–cell interaction and trafficking, and movement are affected. (*N* = 6/group). TPM, total particular matter and TPP1, telomere protection protein 1.

Based on the molecules affected pathway networks affected in TPP1 Flox and CreCC10 mice were determined (Figure 12). Two major networks identified. In TPP1 Flox mice, affected pathways included Cellular movement, inflammatory response, and organismal injury and abnormalities (Figure 12Aa), where, ALDH3A1, CD68, CHI3L1, Chil3/Chil4, CTSD, CTSK, ITGAX, LIPA, LSS, NQO1, PIGR, and THEMIS2 were upregulated while FHL2 and MB were downregulated.

In Network 2, cell‐to‐cell signaling and interaction, cellular movement, and hematological system development and function (Figure 12Ba), where, ARG2, ATP6V0D2, CYP1A1, CYP1B1, PSAP, SPP1, and FCGR2B were upregulated while ALOX15, BPIFA1, and CDH2 were downregulated.

In TPP1 CreCC10, network 1 affected were inflammatory response, lipid metabolism, organismal injury, and abnormalities (Figure 12Ab) where ARG2, ATP6V0D2, CD68, Chil3/Chil4, CLEC7A, CTSD, CTSK, CTSS, CYP1A1, CYP1B1, FABP5, ITGAX, JCHAIN, LIPA, LSS, MSR1, PSAP1, THEMIS2, and VNN1 were significantly upregulated while FABP4 and SERPINA1 were down regulated.

In network 2, Cellular function and maintenance, cellular movement, and immune cell trafficking (Figure 12Bb) where ADGRE1, CHI3L1, Chil3/Chil4, CTSS, IL1RN, LY75, NAPSA, NQO1, PIGR, SFTPD, SFTPD, and SPP1 were upregulated while CDH2, CEBPB, FHL2, and MAVS were downregulated.

### 3.13 Smoke exposure affected more biological functions in TPP1 CreCC10 mice compared to TPP1 Flox mice

**FIGURE 13:**
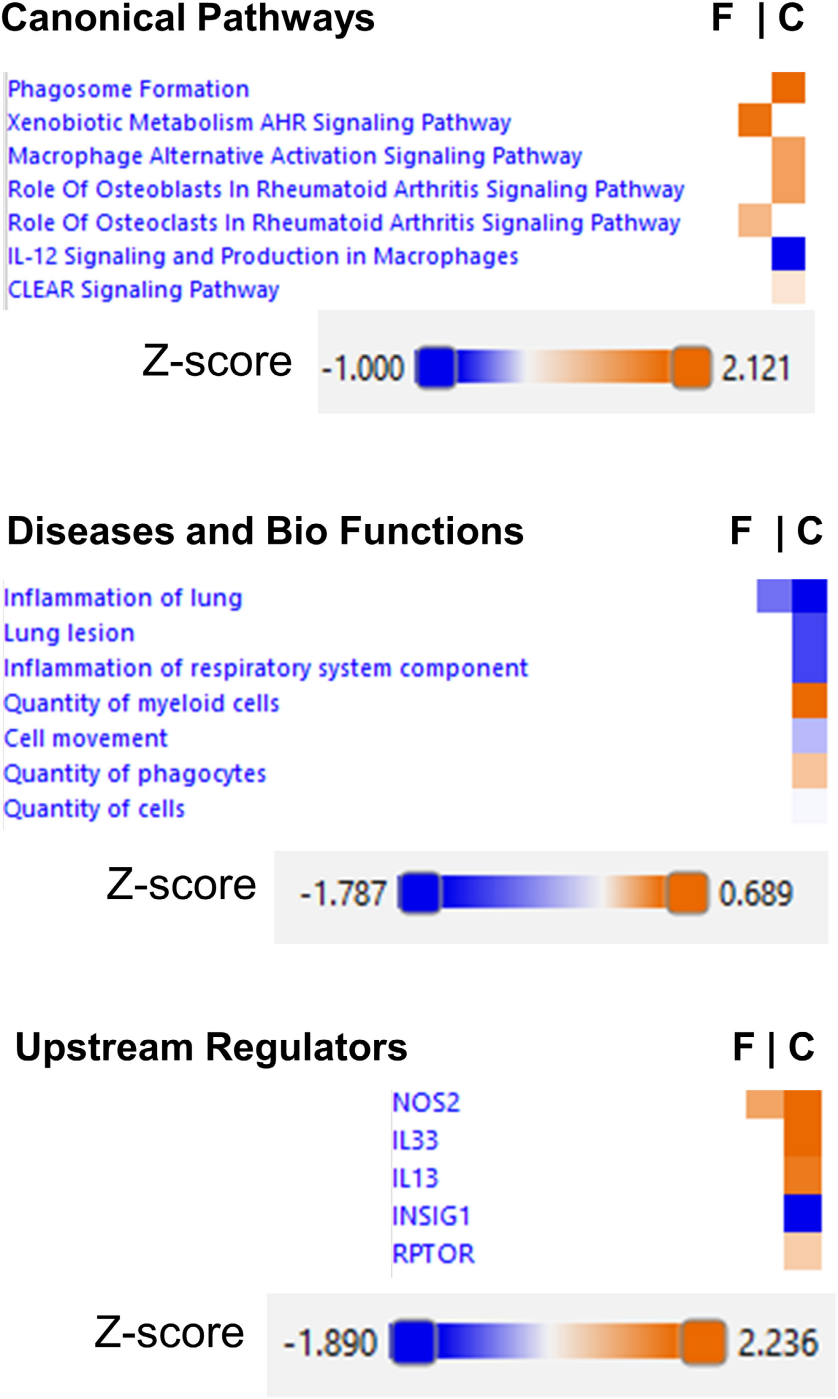
Canonical pathways, diseases and bio functions, and upstream regulators affected in TPP1 CreCC10 mice compared to TPP1 Flox mice. TPP1‐Flox (F) and TPP1‐CreCC10 (C) mice exposed to mainstream smoke (~200 mg/m TPM; 4 months). After 24 h postexposure, mice were euthanized and proteomics were performed on lung tissues. Based on affected molecules in both Flox and creCC10 mice, affected pathways and related diseases were depicted based on Z‐scores (*N* = 6/group). TPM, total particular matter and TPP1, telomere protection protein 1.

Log_2_ fold changes between mainstream smoke‐exposed and unexposed TPP1 Flox and TPP1 CreCC10 mice were analyzed for molecules, bio functions, diseases, and disease pathways according to Z‐scores (Figure 13). In TPP1 CreCC10 mice, the primary canonical pathways affected were phagosome formation, macrophage‐signaling pathways, IL12 signaling, and CLEAR signaling pathway. Affected diseases and biofunctions in TPP1 CreCC10 mice included inflammation of the lung, lesions, and inflammation of respiratory system component, quantity of myeloid cells, cell movement, quantity of phagocytes, and quantity of cells. Identified upstream regulators included NOS2, IL13, INSIG1, and RPTOR in TPP1 CreCC10 mice. In TPP 1 flox mice, xenobiotic metabolism AHR signaling, inflammation of the lung, and NOS2 were altered.

### 3.14 Smoke exposure‐induced abnormalities in TPP1 CreCC10 mice

**FIGURE 14:**
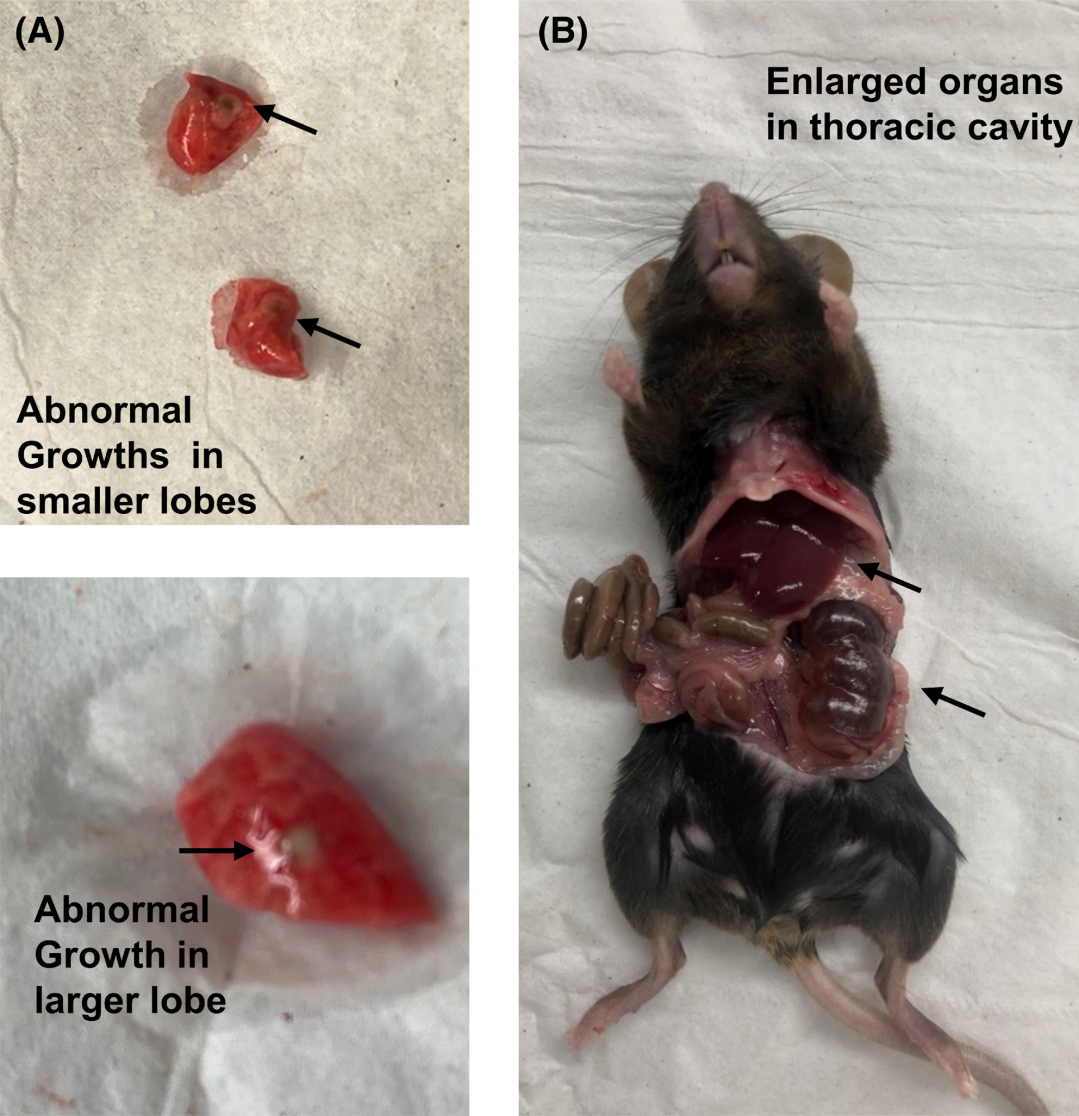
Representative abnormalities observed in TPP1 CreCC10 mouse thoracic cavity. TPP1‐Flox and TPP1‐CreCC10 mice exposed to mainstream smoke (~200 mg/m TPM; 4 months). After 24 h postexposure during euthanasia fluid buildup, enlarged organs, and tumor like growths in lungs and salivary glands were observed. (A) Tumor growth in lungs and (B) enlarged organs in thoracic cavity. TPM, total particular matter and TPP1, telomere protection protein 1.

In smoke‐exposed TPP1 CreCC10 mice, several abnormalities were found during the sacrifice. These include gray‐colored tumor‐like growths in the lungs (Figure 14A) and enlarged organs such as the liver and colon (Figure 14B).

## 4 DISCUSSION

Telomeres play a critical role in preventing the progressive shortening of chromosomes during mitosis. Genomic integrity in chromosomes is maintained by the forces of the shelterin complex, telomerase complex, and CST complex, which are involved in end protection, elongation, and capping.^20^ In this study, we evaluated the effects of conditional removal of TPP1 in clara cells using a surrogate mouse model (TPP1^CreCC10^) compared to their wildtype TPP1 Flox (TPP1 ^fl/fl^) upon exposure to mainstream tobacco smoke, otherwise known as CS. We hypothesized the removal of the shelterin protein, TPP1, would induce shelterin complex dysfunction and cellular senescence, hindering the primary roles of club cells in pulmonary function. We postulated dysregulated functioning of club cells upon exposure to CS, relying on Club cell protein 10 (CC10) secreted along the tracheobronchial tree, particularly in the terminal bronchioles where club cells are localized, a marker for cellular integrity with a pivotal role in detoxifying xenobiotic, control inflammation, mucociliary clearance, surfactant apo‐proteins, protection of the respiratory tract against oxidative stress and inflammation.

Upon chronic smoke exposure, the BALF inflammatory response elicited by both TPP1 Flox and TPP1 CreCC10 mice were similar except for regulated upon activation T cell expressed and secreted (RANTES) that was significantly increased in TPP1 CreCC10 mice. Increased RANTES levels have been observed during airway eosinophilia in asthma and COPD patients along with increased nitric oxide synthase (NOS_2_).^21^, ^22^ This is consistent with the NOS_2_ activation we observed in upstream regulators in disease network pathway analysis. Further, elevated RANTES expression has been observed in patients with lung adenocarcinoma.^23^, ^24^ RANTES is involved in the recruitment of CD4 lymphocytes and CD4‐mediated immune responses via CCR receptors in the adaptive phase.^22^, ^25^ Our data suggest that the removal of TPP1 in club cells may have dysregulated this response.

In acute exposure, we did not observe this with RANTES; rather, we observed a subdued RANTES level in the CreCC10 compared to the Flox group. The 10‐day acute exposure depicted a significantly increased IL6 and a nearly significant G‐CSF response, differing from the chronic exposure. The increase in G‐CSF could be a result of the innate immune response switching over to the adaptive system at the 10‐day, especially on the monocyte/macrophage system by expansion and enhancement of phagocytosis to regulate Th1 mediated cytokine production.^26^

Lung function parameters suggested mild restrictive (fibrosis) phenotype with decreased compliance increased elastance in TPP1 CreCC10 mice concomitantly with increased mild increase in collagen deposition. Our data suggest that the lung may be undergoing mild structural modifications without affecting lung mechanics under these experimental conditions. As the TPP1 mouse strains were middle aged (adult to older), we further investigated age‐related effects of lung function in wildtype mice. Interestingly, we observed age‐dependent lung functional changes in wildtype C57BL/6J mice. While the young (~2 month) did not show any changes in lung function upon CS exposure compared to their counterpart air group, 6 month‐old mice showed a trend toward decreased compliance and increased elastance, coinciding with the observations made in our chronic TPP1 CreCC10 exposed cohort. However, this trend was reversed at 9 months, leaning toward obstructive tendencies, with increased compliance and decreased elastance and alveolar energy dissipation (tissue damping). Our data suggest that lung function decline and pathogenesis are complex processes with age‐dependent effects associated with the immune response.

Smoke exposure caused reduced expression of shelterin complex protein, TRF1. We also identified downregulation of Terf2 gene expression and proteins, similar to human lungs.^5^ These findings suggest the destabilizing of the shelterin complex, thus disrupting t‐loop formation and end protection. We found OBFC1 (STN1) and CTC1 units unaffected by smoke exposure, which is consistent with our proteomics data, suggesting only the end‐capping may not have been affected.

Postexposure to smoke, increased levels of telomere associated zinc finger protein (TZAP) levels were seen in TPP1 CreCC10 mice. TZAP binds to long telomeres with low shelterin protein levels, by competing with TRF1 and TRF2.^27^ Corroborating our data, existing research suggests overexpression of TZAP associated with dysfunctional telomeres and poor prognosis in adenocarcinomas.^28^

We further observed alterations in checkpoint kinases (Chek) 1 and 2. These are involved in fundamental cellular functions such as DNA replication and cell cycle progression, chromatin restructuring, and apoptosis. Chk1 and Chkk2 have many downstream targets involved in cell cycle arrest including Cdc25c, which also was dysregulated in our model upon smoke exposure.^29^ BUB1B mitotic checkpoint protein was significantly elevated in TPP1 CreCC10 mice compared to the reduced expression in TPP1 Flox mice. BUB1B has been identified as a molecular target for lung adenocarcinoma regulating anchorage‐independent growth, anoikis, and metastasis by research studies.^30^

Compared to TPP1 Flox mice, TPP1 CreCC10 mice showed significantly upregulated replication factor C 3, 4, and mini‐chromosome maintenance components 2, 4, and 5 after smoke exposure. These units of the replisome complex are shown to be amplified and correlated with NSCLC and metastases.^31^ Our data suggest that uncontrolled DNA replication may lead to carcinomas in cells without TPP1.

Retinoblastoma protein (Rb), a tumor suppressor protein blocking S‐phase entry is often found in lung cancer cells.^32^ In both TPP1 Flox and CreCC10 strains, we noticed a significant downregulation of Rb. Further, in support, RBL2, RB transcriptional core repressor like2, involved in chromatic binding was downregulated. Inversely, as anticipated, Rb inhibiting p16 (Cdkn1a) was significantly downregulated in both strains. Further, DNA damage repair genes, excision repair (ERCC) 1,2,4,5,6 expression and X‐ray Repair Cross Complementing (XRCC) 1,4,5, and 6 were dysregulated in both TPP1 Flox and CreCC10 post exposure to chronic tobacco smoke. These markers have been studied for their polymorphisms in smoke induced lung cancer.^33^, ^34^, ^35^

The MRE11‐RAD50‐NBS1 complex has a pivotal role in the DNA damage response, replication fork collapse and telomere dysfunction and is known to have lung cancer therapeutic value.^36^ Upon exposure to tobacco smoke TPP1 CreCC10 mice had significant alterations in Mre11 and RINT1 (Rad50 interactor 1) levels, suggesting TPP1 ablation may induce malignancies.

Tumor suppressor p53 mutations and abundance are hallmarks of lung cancer. Its sub‐components and upstream and downstream regulators can emulate similar adverse effects.^37^, ^38^, ^39^ Compare to the TPP1 Flox mice in the TPP abrogated counterparts, Tp53bp1, Tp53bp2, and Ppp1r14b, were significantly downregulated, while Tp53rk (p53 binding activity) was significantly upregulated. These data suggest that polymorphisms, mutations, and dysregulation of p53‐related genes in TPP CreCC10 mice upon exposure to tobacco smoke. BAX, BCL2 associated apoptosis regulator, is regulated by the tumor suppressor p53 and has been shown to be involved in p53‐mediated apoptosis.^40^ p53 associated BCL2 family of proteins, Bnip3l, Bnip2, Bclaf1, and Bak1 were significantly altered in TPP1 CreCC10 mice exposed to smoke compared to the TPP1 Flox mice, implicating the tumorigenic susceptibility due to TPP1 removal. These effects were further supported by anti‐tumorigenic semaphorin III, sema3f downregulation in TPP1 CreCC10 mice.

Identified upstream regulators IL13 and IL33 are consistent with smokers' lungs with excessive mucus secretions.^41^ IL13 is a stimulator of eosinophils, lymphocytes, and macrophage‐mediated inflammation where mucus metaplasia and tissue fibrosis are often seen.^42^ Alternatively, activated macrophages expressing IL13 and Th2 associated IL33 mediators which signal through the ST2 receptor are also associated with bronchial epithelial repair.^43^ We observed over‐expression of Il1rn in TPP1 CreCC10 mice after smoke exposure, which is seen in abnormally activated macrophages.^43^ Il1rn expression may be protecting against lung injury caused by smoke exposure by blocking fundamental proinflammatory cytokines such as TNFα and IL1, as seen in BALF.

Identified canonical pathways exhibited osteoblast and osteoclast dysregulation post‐exposure to tobacco smoke in TPP1 CreCC10 mice. Insulin‐like growth factor 1(IGF‐1) regulates osteoblast function and is involved in lung development. IGF‐1 dysregulations have been associated with alveolar hyperplasia and lethal neonatal respiratory distress.^44^ Further, IGF1 has been identified as a senescence inducer in IPF by releasing CTFG, TGFβ, and MMP9.^45^ This IGF‐mediated tumorigenesis and its role in senescence may be correlated with the increased IGF1 expression observed in TPP1 CreCC10 mice.

Flap endonuclease, FEN1, participates in DNA replication and base excision repair pathways. FEN1 helps maintain genome integrity. TPP1 CreCC10 mice showed upregulated expression of FEN1, which is consistent with the findings that they interact with shelterin proteins TRF2 and TERT.^46^, ^47^ While we can expect increased FEN1 levels as a compensatory mechanism, this may suggest increased tumorigenesis.

Cyclin E1, CCNE1, was significantly increased in TPP1 CreCC10 mice. CCNE1, the regulator of CDK, is amplified in malignant growths in 7.5% of tumor types.^48^ These data suggest that CCNE amplification may be associated with malignancies we observed in TPP1 CreCC10 mice.

Our protein profiling showed increased ALOX5AP and PTPRC expression in lung tissues suggest the presence or the differentiation of basal cells into tuft cells.^49^ 5‐lipoxygenase is for leukotriene synthesis and metabolism. Increased levels are seen in asthma. PTPRC plays a role in cell growth, differentiation, mitosis, and oncogenic transformation.

As the shelterin disruption by TPP1 removal from clara cells demonstrated metabolic telomere dysfunction, polymorphisms in DNA damage repair genes, and telomere dysfunction, tumor proliferative markers such as proliferating cell nuclear antigen (PCNA) and Ki67 were assessed. Significantly increased expression of PCNA and Ki67 in TPP1 CreCC10 corroborates with a high index of these proteins in human lung adenocarcinoma.^38^

Protease cathepsin S is known to contribute to acute respiratory distress syndrome. Consistent with this, in TPP1 CreCC10 mice, increased levels of cathepsin S were observed. This may suggest TPP1 CreCC10 mice are more susceptible to protease‐induced lung injury.^50^

We observed significantly elevated surfactant proteins Sftpd and Sftpa1 and activation MMP9 in those in pathway networks. Surfactant protein A plays a key role in immunoregulation.^51^ Surfactant protein D has been shown to play a protective role against emphysema and other metalloproteinase‐related lung injuries.^52^, ^53^

Overall, our data show conditional ablation of TPP1 in clara cell caused that disruption of shelterin complex by altering the stoichiometry of TRF1 and TRF2, which dysregulated pivotal roles played by clara cells upon exposure to extraneous insults such as tobacco smoke. These dysregulated pathways included xenobiotic phase‐1 metabolism by affecting CYP proteins such as CYP1A1. Phase 2 metabolism via glutathione and glutathione S‐transferases are key for the detoxification of xenobiotic and to ameliorate oxidative stress.^54^, ^55^, ^56^, ^57^ The reductions in Glutathione S‐transferase 1, 2, 5, and 7 observed in TPP1 CreCC10 suggest increased reactive carcinogens promoting carcinogenic effects.

Further, significant changes in IL‐13, ‐33, and ‐12 pathways were observed along with phagosome alterations and lung injury response defects. Changes in biomolecules and transcription factors increased susceptibility of squamous cell neoplasms and adenocarcinomas. Further, in middle age (~6–7) wild type (C57BL/6J) and TPP1 Flox/Cre mice showed similar lung function decline, suggesting CS‐induced lung damage and decline in function may be age‐dependent. In summary, our data suggest disruption of the shelterin complex may increase susceptibility to lung diseases such as COPD, fibrosis, tumorigenesis/cancer upon exposure to environmental toxicants.

In conclusion, TPP1 plays a protective role against lung inflammation, injury repair, COPD and restrictive lung function decline, DNA damage, dysregulated phase 1 and 2 metabolism and carcinogenesis. Overall, TPP1 plays an important role in maintaining lung homeostasis and injurious responses with age‐dependent effects in response to CS in chronic lung diseases and COPD/emphysema.

## AUTHOR CONTRIBUTIONS

T.M. and I.R. Conceived and designed the experiments. TM performed the experiments and data analysis, CG performed trichrome staining, and analyzed data. TM, CG, and IR wrote the manuscript. TM, CG, and IR edited the manuscript.

## FUNDING INFORMATION

This work was supported by the National Institutes Health: NIH R01 HL135613, R01 ES029177, R01 HL13773, HL158316‐A1, HL167655‐A1, and HL147715‐01 by IR, and K99/R00 ES033835 by Thivanka Muthumalage.

## CONFLICT OF INTEREST STATEMENT

The authors declare that the research was conducted in the absence of any commercial or financial relationships that could be construed as a potential conflict of interest, and no potential conflicts of interest with respect to the authorship, and/or publication of this article.

## DISCLAIMER

The authors have nothing to claim or disclaim about any products used here to test their toxicological and biological effects.

## Supporting information


Data S1:

## Data Availability

We declare that we have provided all the data, but the primary data will be available upon request.
