# Supplementary material for: Club cell‐specific telomere protection protein 1 (TPP1) protects against tobacco smoke‐induced lung inflammation, xenobiotic metabolic dysregulation, and injurious responses
Source: FASEB Bioadv. 2024 Jan 15;6(2):53–71. doi: 10.1096/fba.2023-00115 (PMC10853660; doi:10.1096/fba.2023-00115)

Supplementary Figures

Club cell-specific telomere protection protein 1 (TPP1) protects against tobacco smoke-induced lung inflammation, xenobiotic metabolic dysregulation, and injurious responses

Thivanka Muthumalage, Chiara Goracci, and Irfan Rahman

[1]Department of Environmental Medicine, School of Medicine and Dentistry,

University of Rochester Medical Center, Rochester, NY, USA

Supplementary Figure 1:

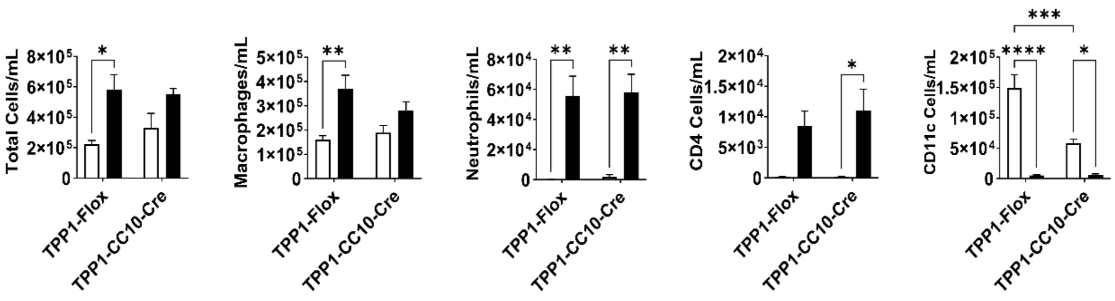

Supplementary Figure 1. **TPP1 does not protect against of acute CS-exposure induced inflammatory cell influx in bronchoalveolar lavage (BAL) fluid**. TPP1-flox and TPP1-CC10-Cre mice were exposed to CS for 10 days. Differential cell counts were analyzed using BAL fluid and count of inflammatory cells (F4/80+ macrophages, LY6B.2+ neutrophils, CD4+ T-lymphocytes and CD11c+ dendritic cells) was determined by flow cytometry. Cells from BAL fluid were stained with AO/PI to count total number of cells using cellometer. Data are shown as mean ± SEM (n= 5/group). Significance determined using Two- way ANOVA followed by Tukey’s multiple comparisons test. * P < 0.05, ** P < 0.01, *** P < 0.001, **** P < 0.0001, respective air exposed control groups and indicated groups.

Supplementary Figure 2:

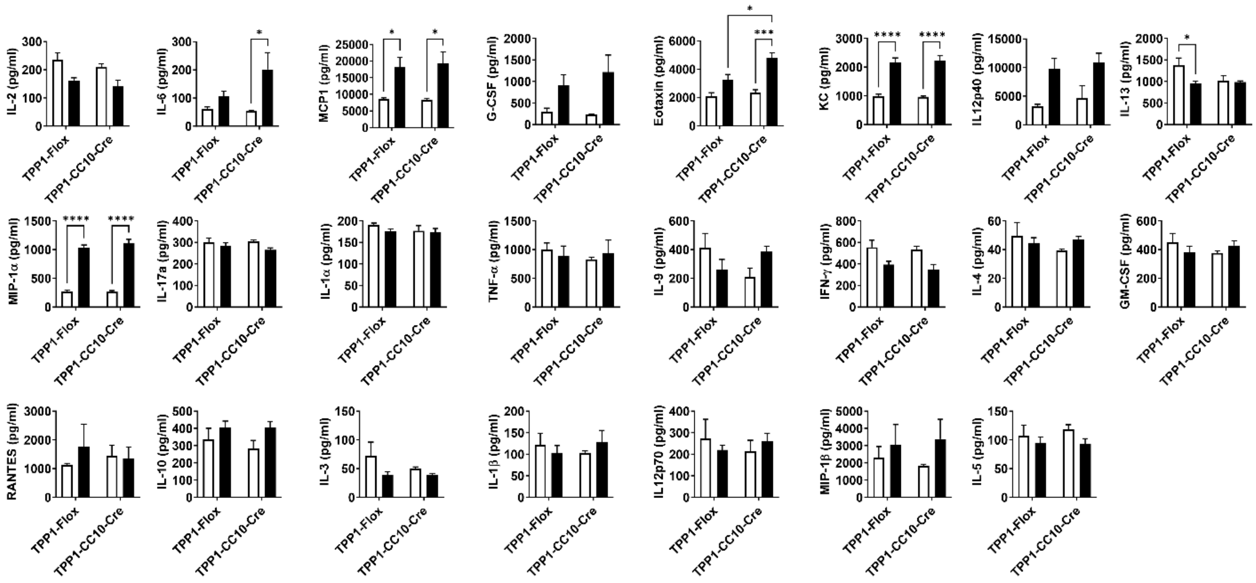

Supplementary Figure 2: TPP1 does not mitigate the inflammatory cytokine response elicited by acute CS exposure. TPP1-Flox and TPP1-KO mice were exposed to CS for 10 days and BAL fluids were used to determine SASP cytokines. (A) The level of proinflammatory cytokines measured using luminex multiplex assay. Data are shown as mean ± SEM (n = 5/group). Significance determined using 2-way ANOVA followed by Tukey's multiple comparisons test. * $P < 0.05$, *** $P < 0.001$, **** $P < 0.0001$ vs. Air and indicated groups).

Supplementary Figure 3:

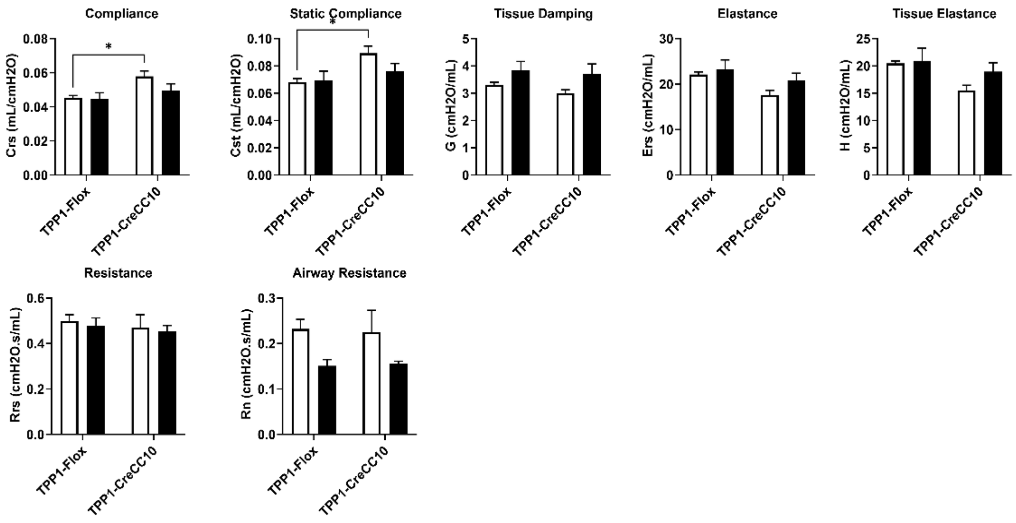

Supplementary Figure 3: Exposure to mainstream tobacco smoke did not alter lung mechanics parameters in older TPP1 Flox mice with marginally increased elastance in TPP1 CreCC10 mice.

Older (up to15 months) TPP1-Flox and TPP1-CreCC10 mice exposed to mainstream smoke (~200 mg/m TPM; 4 mo). After 24h post-exposure, mice were anesthetized and lung function parameters including, compliance, elastance, resistance and . tissue damping were measured by Flexivent. (N = 6–8/group, *P < 0.05 vs. Air and indicated groups)

Supplementary Figure 4:

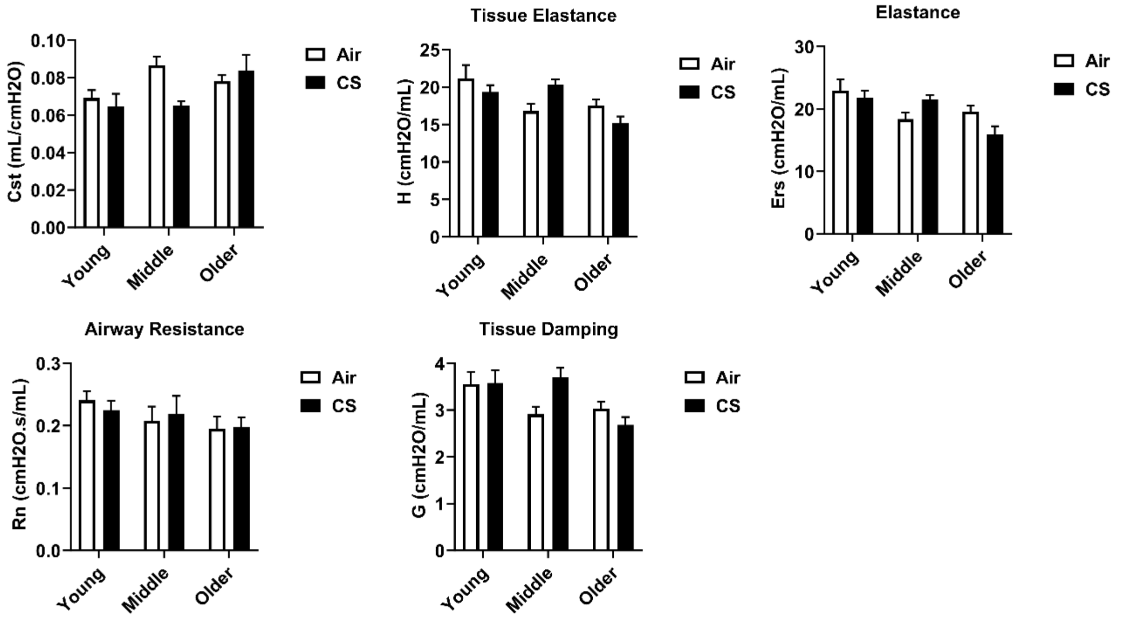

Supplementary Figure 4: Lung mechanics altered in an age-dependent manner in C57BL/6J upon exposure to CS.

C57BL/6J mice of three different age groups, 2 months (young), 6 months (middle), and 9 months (older) were exposed to mainstream tobacco smoke for 5 months (2h/day, 5d/week) at 200 mg/m3 TPM. Lung compliance, elastance, airway resistance, and tissue damping were measured in CS exposed and air control group upon euthanasia and compared between the three age groups, young, middle, and older. (N = 6–8/group)

Supplementary Figures 5-8: Full western blots of Chk1, OBFC1, TRF1, and TZAP with loading control, β actin, used for the normalization of target protein as shown in Fig. 5. For Chk1, OBFC1, and TRF1, the bands of interest were normalized to β-actin of the same blot. TZAP was normalized to β-actin from corresponding samples due to a nonspecific band in the region of loading control.

__Supplementary Figure 5__

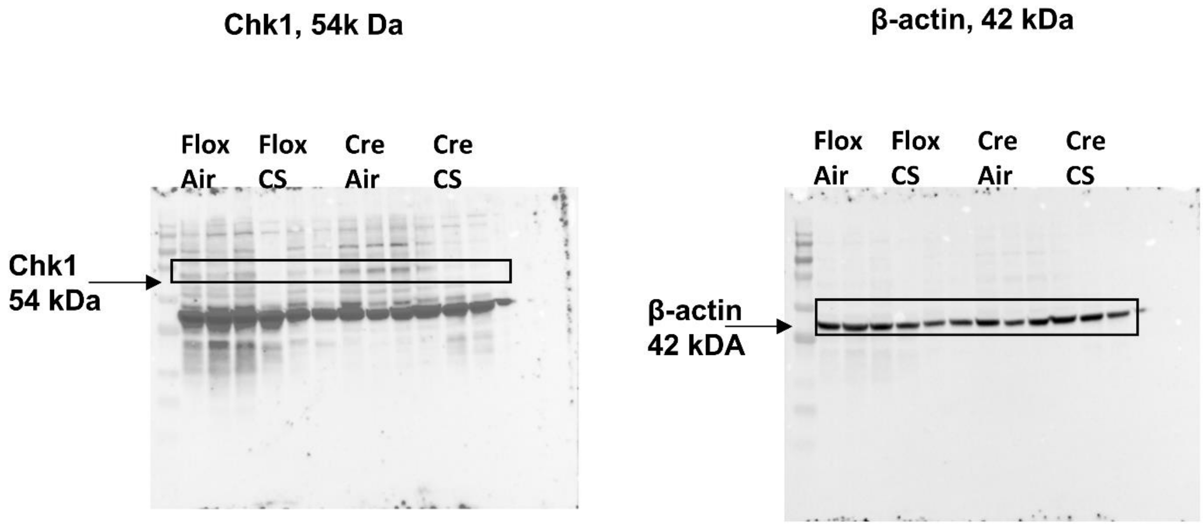

__Supplementary Figure 6__

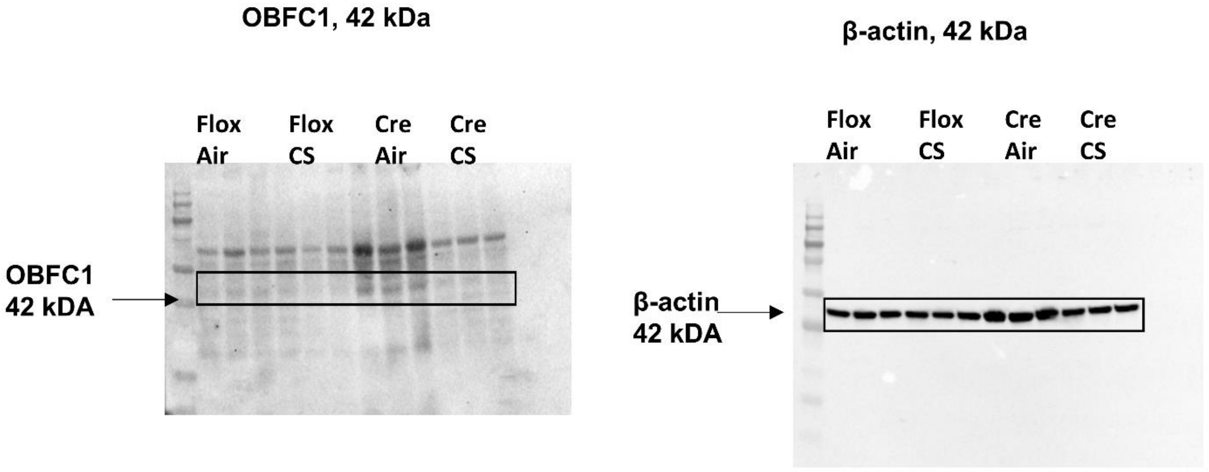

Supplementary Figure 7

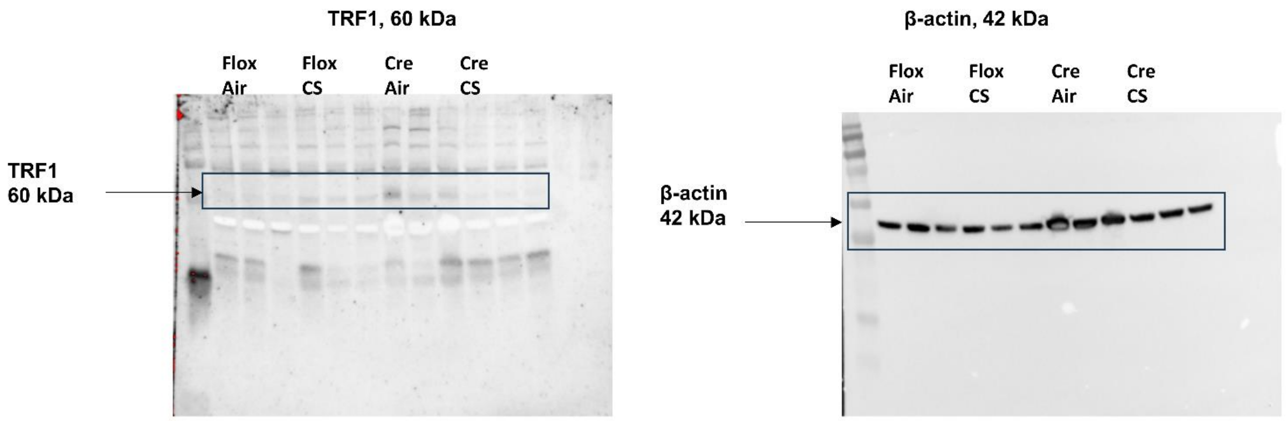

Supplementary Figure 8

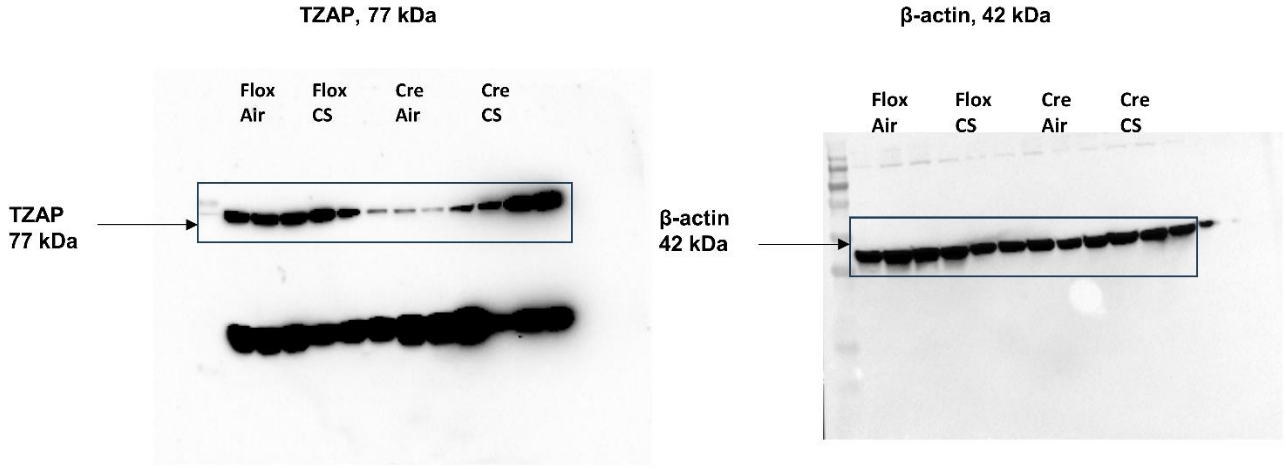

Supplement: Supplementary file 1 — Data S1: [file FBA2-6-53-s001.pdf]
